# A Neutral “Aluminocene” Sandwich Complex: η^1^‐ versus η^5^‐Coordination Modes of a Pentaarylborole with ECp* (E=Al, Ga; Cp*=C_5_Me_5_)

**DOI:** 10.1002/anie.201907749

**Published:** 2019-09-10

**Authors:** Christian P. Sindlinger, Paul Niklas Ruth

**Affiliations:** ^1^ Institut für Anorganische Chemie Georg-August-Universität Göttingen Tammannstrasse 4 37077 Göttingen Germany

**Keywords:** aluminocenes, aromaticity, boroles, Group 13 elements, main group complexes

## Abstract

The pentaaryl borole (Ph*C)_4_BXyl^F^ [Ph*=3,5‐*t*Bu_2_(C_6_H_3_); Xyl^F^=3,5‐(CF_3_)_2_(C_6_H_3_)] reacts with low‐valent Group 13 precursors AlCp* and GaCp* by two divergent routes. In the case of [AlCp*]_4_, the borole reacts as an oxidising agent and accepts two electrons. Structural, spectroscopic, and computational analysis of the resulting unprecedented neutral η^5^‐Cp*,η^5^‐[(Ph*C)_4_BXyl^F^] complex of Al^III^ revealed a strong, ionic bonding interaction. The formation of the heteroleptic borole‐cyclopentadienyl “aluminocene” leads to significant changes in the ^13^C NMR chemical shifts within the borole unit. In the case of the less‐reductive GaCp*, borole (Ph*C)_4_BXyl^F^ reacts as a Lewis acid to form a dynamic adduct with a dative 2‐center‐2‐electron Ga−B bond. The Lewis adduct was also studied structurally, spectroscopically, and computationally.

## Graphical abstract

**One way or another**: Two divergent routes of a weakly anti‐aromatic and Lewis‐acidic pentaarylborole with the monolvalent Group 13 cyclopentadienyl compounds AlCp* and GaCp* have been identified. The energetic accessibility of their two lone pairs of electrons determines whether a redox reaction to form a neutral heteroleptic borolediide/cyclopentadienyl “aluminocene” or formation of a Lewis‐base adduct with a dative Ga−B bond is observed.

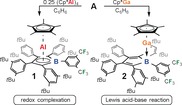

Fifty years ago, Eisch reported the first authentic isolation of pentaphenyl borole.^1^ Free boroles are weakly anti‐aromatic cyclic 4π‐electron compounds.^2^ Among a variety of intriguing reactivities, including the activation of hydrogen^3^ or Si−H bonds,^4^ Diels–Alder reactions, and ring expansions,1b, 5 boroles can be readily reduced by two electrons to form Hückel‐aromatic borolediides^6^ or they can react as potent Lewis acids.^7^ In recent years, variation of the boron‐bound substituent allowed for an extension of the library of known boroles with substantially altered optical gaps.2b, 6b, 8

The coordination chemistry of boroles toward transition metals has been studied since the late 1970s.6a, 9 However, despite the isoelectronic nature of borolediide with the—in organometallic chemistry—ubiquitous and iconic cyclopentadienyl anion, very few complexes other than with d‐block metals or very electron‐positive s‐block metals are known. Recently Müller, Albers, and co‐workers reported a Ge^II^‐borole complex that resulted from a rearrangement during the reaction of a germole dianion with amidoborane dihalides.^10^ Although only a few comments are found in the literature,9d, 11 a likely reason for the scarcity of p‐block complexes, in particular, is that borolediide salts act as reducing agents rather than as a ligand source in metathesis reactions with p‐block halides.

We recently reported the synthesis of a set of novel, highly soluble *tert*‐butyl‐decorated pentaphenyl boroles (Ph*C)_4_BR [Ph*=3,5‐*t*Bu_2_(C_6_H_3_)].^12^ We are interested in further expanding the chemical scope of boroles as ligands to the p‐block elements. To circumvent salt metathesis reactions, we treated borole (Ph*C)_4_BXyl^F^ (**A**) with the established, potentially reductive monovalent Group 13 reagents (AlCp*)_4_ and GaCp* (Scheme 1).

**Scheme 1:**
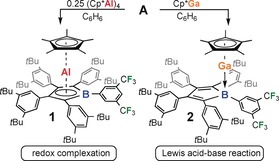
Divergent reaction pathways of free borole **A** with AlCp* and GaCp*.

When GaCp* was added to borole **A** an immediate colour change from dark green to bright orange was observed. NMR spectroscopic examination of the reaction mixture confirmed a clean conversion and the formation of a single product. The ^1^H NMR spectrum revealed no substantial changes in the shifts compared to the individual starting materials. However, the ^11^B NMR signal drastically shifts from a broad signal in the typical range of tricoordinate boron atoms at *δ*_11B_=71 ppm (ω_1/2_=ca. 3250 Hz) in **A** to a narrower signal at *δ*_11B_=7.6 ppm (ω_1/2_=ca. 1550 Hz) in **2**. The shift to higher field is a clear indication of a higher coordination number at the boron atom.^13^ Major changes (≥±2 ppm) in the ^13^C{^1^H} NMR spectrum of the borole framework are observed for the α‐ and β‐carbon atoms of the C_4_B cycle as well as the *ipso*‐ and *para*‐positions of the boron‐bound aryl moiety (Table 1).

**Table 1:** Diagnostic NMR chemical shifts in C_6_D_6_ at 298 K of **A**, **1**, and **2**. Calculated averaged values in brackets.

Compound	C_β_^[b]^	C_α_^[b]^	*i*‐C_XylF_^[b]^	*p‐*C_XylF_^[b]^	^11^B
**A** ^[a]^	166.2	140.6	135.9	125.3	71.6
**1**	128.4 [126.1]	118.0 [117.9]	144.2 [144.8]	119.1	24.6/17.3^[c]^ [18.6]
**2**	151.2 [151.7]	149.6 [149.9]	150.7 [151.6]	119.4	7.6/−0.4^[d]^ [−0.9]

[a] See Ref. 12. [b] ^13^C NMR shift in ppm in C_6_D_6_. [c] At −75 °C in toluene. [d] At −50 °C in toluene.

An interaction of the GaCp* fragment with the boron‐centred LUMO is also in line with the change in colour from an intense green (stemming from π/π* excitation in free boroles) to a bright orange. The colour of **2** is unique among the otherwise colorless (Cp/R)Ga^I^ adducts with Lewis‐acidic boranes.^13^, ^14^

At ambient temperature, no further signal for free GaCp* was observed after addition of a further 0.5 equiv of GaCp* to solutions of **2**, thus indicating a dynamic exchange of GaCp*. Variable‐temperature NMR experiments of solutions of **2** in toluene with a slight excess of GaCp* reveal hindered rotation of the C_β_‐bound Ph* groups starting at −40 °C. At −30 °C, the Cp* signal significantly broadens and gradual cooling from −40 °C to −75 °C leads to two increasingly sharp separate Cp* signals of GaCp* and **2** being observed. The ^1^H NMR chemical shifts all lie in the range of pure GaCp*, which is reported to likely form hexamers at low temperature.^15^ However, the intense orange colour does not change upon cooling, thus rendering a potential equilibrium between **2** and **A**+1/6 [GaCp*]_6_ unlikely. Orange‐red crystals suitable for X‐ray diffraction grew from benzene solutions. The molecular structure clearly confirms the formation of a boron‐centred Lewis‐base adduct, with donation of the Ga^I^ lone pair of electrons into an empty p orbital on boron (Figure 1). The Ga1–Cp*_centroid_ vector is virtually aligned with the Ga1−B1 bond (175.5°), and the Ga1–B1 vector is almost perpendicular to the C_4_B plane (C4‐B1‐Ga1 95.04(11)°, C1‐B1‐Ga1 92.60(11)°. The Ga−B bond (2.1382(19) Å) is similar to those in B(C_6_F_5_)_3_ adducts of GaCp derivatives (2.154(3), 2.155(6), 2.161(2) Å).13a, 14b The bond lengths within the borole ring clearly reveal isolated C=C and C−C bonds. The Xyl^F^ residue at the tetracoordinate boron centre noticeably bends out of the borole plane away from the GaCp* cone. A related structural motif and reactivity was also observed for AlCp* adducts of 9‐borafluorenes.^11^

**Figure 1:**
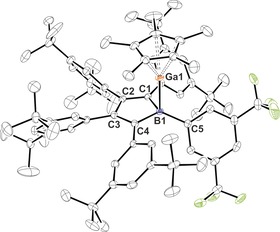
ORTEP plot of the molecular structure of the Lewis acid‐base complex (**A**⋅GaCp*) (**2**).^26^ Atomic displacement parameters are drawn at the 50 % probability level. Hydrogen atoms, disordered *t*‐Bu groups, and a lattice benzene molecule have been omitted for the sake of clarity. Selected bond lengths [Å] and angles [°] are given: Ga1‐B1 2.1382(19), B1‐C5 1.599(3), B1‐C1 1.604(3), B1‐C4 1.600(3), C1‐C2 1.370(2), C2‐C3 1.471(2), C3‐C4 1.377(2), Ga1‐Cp* 2.2152(18), 2.2355(19), 2.2579(18), 2.2754(19), 2.2973(19), Ga1‐Cp*_centroid_ 1.902; C5‐B1‐Ga1 109.28(12), C4‐B1‐Ga1 95.04(11), C1‐B1‐Ga1 92.60(11), B1‐Ga1‐Cp*_centroid_ 175.5.

Over the course of a few weeks, small amounts of a fine grey solid deposited from solutions of **2** along with the formation of unassigned decomposition products.^15^

Clearly, the monovalent Ga^I^Cp* was too reluctant to transfer electrons and reduce the borole. We therefore turned to (AlCp*)_4_, as Al^I^ is a stronger reductant. AlCp derivatives can also form base adducts with boranes.^16^ Suspending the poorly soluble (AlCp*)_4_ in green solutions of **A** leads to a very slow decolourisation over the course of three days to finally yield pale yellow solutions. Monitoring the process by NMR spectroscopy revealed a very clean conversion into a single product **1**. Crystals of **1** readily form from concentrated solutions in various hydrocarbons. In all cases, and despite numerous attempts, we obtained poorly resolved diffraction data.^17^ Examination of the data, however, allowed the key structural feature to be clearly identified: the anticipated quasi η^5^‐Cp*,η^5^‐[(Ph*C)_4_BXyl^F^] Al^III^ sandwich complex **1**. This represents the first neutral “aluminocene” and the second borole complex of a p‐block element.^10^, ^18^ Heteroleptic Cp/borole sandwich complexes are known for various transition metals.8e, 19

The quality of the data limits extensive structural discussion; however, some key features can clearly be identified. Compared to **A** and **2**, which both feature localized cyclic 1,3‐butadiene systems, the atomic distances within the (C_4_B) ring in **1** are much more uniform. Shortened B−C_α_ and C_β_−C_β_ bonds together with an elongated C_α_−C_β_ bond are in line with substantial π‐delocalization, as expected for a Hückel‐aromatic boroldiide.6b The Al1‐(C_4_B)_centroid_ distance is approximately 1.80 Å, which is slightly shorter than the Al1‐Cp*_centroid_ distance of approximately 1.86 Å. This is rationalized by greater electrostatic attraction between the dianionic borole and Al^III^ compared to the simple monoanionic Cp*. The Cp* and borole units adopt a distorted staggered conformation. The Cp*‐Al contacts range from 2.17(1) to 2.27(1) Å, thus indicating a slight deviation of the Al atom from an ideal central localisation. The disorder in the X‐ray structure prevents discussion of individual Al1−(C_4_B) distances. The DFT‐optimised structure (Figure 2) reveals a centered Al atom with comparatively short Al−C_α_ and Al−B contacts. All other experimental structural features are in general good agreement with the gas‐phase DFT‐optimised structure.^20^

**Figure 2:**
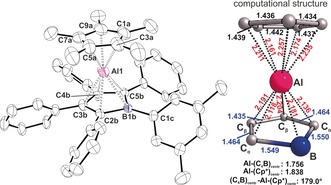
ORTEP plot (left)^26^ and excerpt from the gas‐phase DFT‐optimised^20^ molecular structure of the Al^III^ sandwich complex **1**. Atomic displacement parameters are drawn at the 40 % probability level. Hydrogen and fluorine atoms, *t*Bu groups, and a disorder of ca. 50 % occupation of the borole subunit have been omitted for the sake of clarity. Selected bond lengths [Å] and angles [°] are given. Disorder fraction given in parentheses: B1b‐C2b 1.54(2)[1.54(2)], C2b‐C3b 1.47(2)[1.46(2)], C3b‐C4b 1.41(2)[1.42(2)], C4b‐C5b 1.47(2)[1.47(2)], C5b‐B1b 1.53(2)[1.53(2)], B1b‐C1c 1.59(2)[1.59(1)], B1b‐Al1 2.14(2)[2.31(2)], C2b‐Al1 2.22(1)[2.25(1)], C3b‐Al1 2.32(1)[2.19(2)], C4b‐Al1 2.17(1)[2.12(2)], C5b‐Al1 2.00(1)[2.10(2)], Al1‐C3a 2.27(1), Al1‐C5a 2.22(1), Al1‐C7a 2.17(1), Al1‐C9a 2.18(1), Al1‐C1a 2.24(1); (C_4_B)_centroid_‐Al1 1.77[1.80], Cp*_centroid_‐Al1 1.86; (C_4_B)_centroid_‐Al1‐ Cp*_centroid_ 175.6[174.8].

Complex **1** reveals a ^11^B NMR signal at *δ*_11B_=24.6 ppm, shifted upfield from **A** but less so than **2**. A very broad ^27^Al NMR resonance was observed at *δ*_27Al_=−86.2 ppm (ω_1/2_=ca. 2600 Hz). Both shifts are in good agreement with those predicted computationally for the optimised gas‐phase structure of *δ*=18.6 ppm (^11^B) and *δ*=−90.0 (^27^Al).^21^ The broad ^27^Al resonance is different from the sharp signals in aluminocenium cations and is likely caused by a lower symmetry and the quadrupole moments of the boron nuclei. The ^27^Al chemical shift of **1** lies in‐between those of half‐sandwich complexes, such as (AlCp*)_4_ (*δ*=−78.3 ppm),^22^ (AlCp*)‐η^1^‐9‐Ph‐9‐borafluorenes (*δ*=−70.3 ppm),^11^ or AlCp*‐B(C_6_F_5_)_3_ (*δ*=−59.3 ppm),16a and its closest structural relatives [Cp*_2_Al]^+^ (*δ*=−102.9 ppm), [Cp′_2_Al]^+^ (*δ*=−113 ppm; Cp′=Me_4_C_5_H), and [Cp_2_Al]^+^ (*δ*=−126.4 ppm).^23^ The upfield shift in cationic aluminocenes has been associated with the aromatic nature of the [Cp]^−^ ligands.23a The observed ^27^Al shift for **1** is, therefore, in line with a less pronounced aromaticity of borolediides. The symmetric ^1^H NMR spectrum of **1** recorded in toluene at room temperature barely differs from the spectrum of free borole **A**, which indicates little hindrance of Ph* rotations around the Ph*−C_α/β_ bond. However, cooling readily leads to significant broadening of the *o*‐Ph* signals in both the α‐ and β‐positions. At −15 °C, these signals are broadened beyond recognition and at −75 °C up to eight individual signals for the *o*‐Ph* protons and *t*Bu groups are present, along with a single broad Cp* resonance. This can be rationalized by a static borole subunit structure much like that observed in the solid state with totally locked Ph*‐C_α/β_ rotations that even suppress a switching between the tilt conformation of the borole paddlewheel. This low‐temperature behaviour is significantly different from **2** and strongly supports the η^5^‐(borole) coordination mode being maintained in cool solutions.

The two fundamentally different reaction pathways of borole **A** with GaCp* and AlCp* also become apparent in diagnostic ^13^C chemical shifts of the C_α_‐ and C_β_‐carbon atoms of C_4_B (Table 1). Two‐electron reduction and complexation to form compound **1** results in the rather low‐field‐resonating signals observed in free borole **A** drastically shifting to a higher field by 37.8 ppm (C_β_) and 22.6 ppm (C_α_). Their assignment is supported by excellent agreement with the computationally predicted shifts. This field range is commonly observed for cyclopentadienyl resonances of ECp derivatives. The excellent agreement of the *δ*_calc_ and *δ*_exp_ values also further corroborates the η^5^‐type coordination mode of the borole to be present both in the solid state as well as in solution.

In the case of base adduct **2**, only C_β_ is shifted to a higher field, whereas C_α_ resonates at an even lower field than in **A**. Interestingly, both fundamentally different reactions cause the *p*‐Xyl^F^ resonance to shift to a slightly higher field, which is more typical for *p*‐aryl groups. This is likely due to the population of the empty p‐orbital on boron and prevention of mesomeric delocalization of a positive charge through the π‐system into the boron‐bound aryl group.

Compounds **1** and **2** were also investigated by mass spectrometry using a LIFDI set‐up.^24^ Whereas **1** revealed clean spectra of only [M(**1**)]^+^, concentrated solutions of **2** in toluene under identical conditions revealed only [M(**A**+H_2_O)]^+^ and, to a lesser extent, [M(**A**)]^+^. This is surprising as we never observe [M(**A**)]^+^ in pure solutions of **A**, which always revealed clean [M(**A**+H_2_O)]^+^ signals.

Computational probing of the complexes **1** and **2** allows further insight into the electronic structure of the two different interactions modes of borole (Ph*C)_4_BXyl^F^ (**A**) with ECp* (E=Al, Ga). The computational (BP86‐D3/def2‐TZVP) free dissociation energy to form free **A** and ECp* is substantially higher for **1** (39.4 kcal mol^−1^) than for **2** (12.8 kcal mol^−1^).

The successful transfer of two electrons onto the borole ring in **1** becomes apparent from the borole‐based HOMO essentially being identical with the LUMO in free **A** (Figure 3). LUMO+2 is Al‐based with high s‐character. This is further in line with a Bader charge of +2.29 at Al. The borole (C_4_B) unit accumulates a Bader charge of −0.78. However, this charge resides on the butadiene backbone (C_β_ −0.24; C_α_ −0.99; B +1.68). As expected, the charge accumulated on the central (C_5_)‐Cp* moiety amounting to −1.17 is equally distributed between the five carbon atoms. A QTAIM topology analysis revealed no bond critical point on the Al‐B vector; however, ring and cage critical points are found (Figure 4). In line with a strong localisation of electron density on C_α_, bond critical points are only found for the Al‐C_α_ vectors (delocalization index, DI=0.25) but not for the Al‐C_β_ contact (DI=0.11).^25^ The analysis of the hypothetical model complex (C_4_BH_5_)Al(C_5_H_5_)^11^ revealed identical features. Müller, Albers, and co‐workers also found no Ge−B bonding path in their Ge^II^ aminoborole complex.^10^ Similar Wiberg bond indices (WBI) for all the Al‐(C_4_B) contacts support the η^5^‐coordination mode of the borole (Scheme 2 a). A comparatively high WBI for the C_β_−C_β_ bond is in line with the putatively dominating resonance structure **IV**, which also corroborates the QTAIM charge localization on C_α_. A natural resonance theory (NRT) calculation on the isolated [C_4_BH_5_]^2−^ dianion provides a contribution weighting of resonance structures **I**–**III. IV** is not proposed by NRT, but can be directly derived from **II**. The accumulation of dianionic charge on the C_α_−B−C_α_ moiety (**II** and **III**) accounts for the relatively short B−Al distances observed for the structures of all the computationally probed (C_4_B)AlCp derivatives (Scheme 2 b).

**Figure 3:**
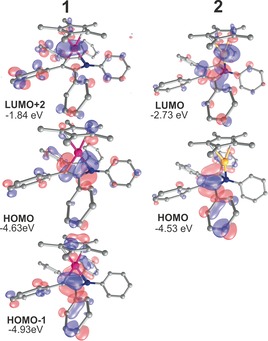
Frontier orbital depictions of molecules **1** and **2**.^20^ *t*Bu and CF_3_ groups are omitted for clarity. Isosurfaces are shown at 0.04 a.u.

**Figure 4:**
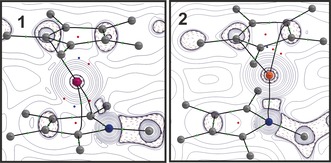
Excerpts of the molecular graph and contour plots of the Laplacian of the electron density (∇^2^ *ρ*(*r*)) isosurfaces through the E‐B‐(C_β_‐C_β_)_centroid_ planes of molecules **1** (left) and **2** (right). Maroon dotted lines: negative Laplacian (area of charge concentration), blue solid lines: positive Laplacian (area of charge depletion), green dots: bond critical points, red dots: ring critical points, blue dots: cage critical points.^20^

**Scheme 2:**
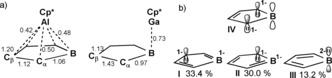
a) WBI for **1** and **2**.^20^ b) Selection of mesomeric descriptions of [C_4_B]^2−^ that putatively contribute to the structure of **1**. The weightings of resonance structures **I**–**III** were obtained from NRT calculations on isolated [C_4_BH_5_]^2−^, with **IV** being a putative dominant resonance structure of **1**.

The HOMO and LUMO in gallium(I) adduct **2** still greatly resemble those in free borole **A**, with the LUMO revealing strong contributions of the GaCp* fragment. The dative Ga−B bond is instead delocalized over several lower lying MOs. A bond critical point was found on the Ga‐B vector and a Bader charge of +0.79 was calculated for Ga. The borole (C_4_B) unit is almost neutral with a combined Bader charge of +0.32 versus an anionic Cp* (C_5_) moiety (−0.73).

In summary, we have presented two divergent routes of a weakly anti‐aromatic and Lewis‐acidic pentaarylborole with monovalent Group 13 cyclopentadienyl compounds, namely AlCp* and GaCp*. Depending on the energetic accessibility of their two lone pairs of electrons, we observed either redox chemistry to form a neutral heteroleptic borolediide/cyclopentadienyl “aluminocene” or formation of a Lewis‐base adduct with a dative Ga−B bond. These observations on the stability and bonding interactions of p‐block complexes of boroles with electropositive p‐block metals improve the understanding of the general applicability of boroles in coordination chemistry.

## Conflict of interest

The authors declare no conflict of interest.

## Supporting information

As a service to our authors and readers, this journal provides supporting information supplied by the authors. Such materials are peer reviewed and may be re‐organized for online delivery, but are not copy‐edited or typeset. Technical support issues arising from supporting information (other than missing files) should be addressed to the authors.

Supplementary
