# Supplementary material for: A Neutral “Aluminocene” Sandwich Complex: η1‐ versus η5‐Coordination Modes of a Pentaarylborole with ECp* (E=Al, Ga; Cp*=C5Me5)
Source: Angew Chem Int Ed Engl. 2019 Sep 10;58(42):15051–6. doi: 10.1002/anie.201907749 (PMC6856865; doi:10.1002/anie.201907749)
Supplement: Supplementary file 1 — Supplementary [file ANIE-58-15051-s001.pdf]

Angewandte Chemie
Eine Zeitschrift der Gesellschaft Deutscher Chemiker

Supporting Information

A Neutral “Aluminocene” Sandwich Complex: η[1]- versus η[5]-Coordination Modes of a Pentaarylborole with ECp* (E = Al, Ga; Cp* = C_5Me_5)

Christian P. Sindlinger and Paul Niklas Ruth*

anie_201907749_sm_miscellaneous_information.pdf

Supporting Information

Table of Contents

Experimental Details S2

General Information S2

NMR spectroscopy S2

Mass spectrometry S2

Starting materials and reagents S2

Synthesis and Analytical Data S3

η^5-[1-(3',5'-Bis-(trifluoromethyl)phenyl)-2,3,4,5-tetrakis(3',5'-di-*tert.*-butylphenyl)-borole]- η^5-Cp*-Aluminocene S3

η^1-[1-(3',5'-Bis-(trifluoromethyl)phenyl)-2,3,4,5-tetrakis(3',5'-di-*tert.*-butylphenyl)-borole]- η^5-Cp*-Gallium S13

Crystallographic Details S23

Data Acquisition and Processing S23

Crystallographic and Refinement Details 1 .... S23

Refinement Details 2 .... S24

Computational Details S25

Structure Optimisation, Frequency Calculation and Thermochemical Approximations S25

Summary GIAO-NMR computations S26

Frontier Orbital Depictions S28

Topology Analyses S28

NBO and NRT Analyses S30

XYZ-coordinates of optimised structures S31

Literature S34

Experimental Details

General Information

All manipulations requiring handling under inert conditions were carried out under argon atmosphere using standard Schlenk techniques or an MBraun Glovebox with an Ar atmosphere. Benzene was obtained from an MBraun SPS and stored over molecular sieves, toluene and ether were distilled from sodium and degassed. Hexane and pentane were distilled from Na/K alloy. THF was distilled from potassium. Benzene-d_6 and toluene-d_8 were distilled from potassium, degassed and stored in a glove box.

Elemental analyses were performed by the Analytisches Labor, Institut für Anorganische Chemie, Universität Göttingen.

NMR spectroscopy

NMR spectra were recorded with either a Bruker Avance III 400 NMR spectrometer equipped with a 5 mm BBFO ATM probe head and operating at 400.13 (^{1}H), 100.61 (^{13}C), 128.38 (^{11}B) and 376.45 MHz (^{19}F) along with a variable temperature set-up or a Bruker Avance Neo 400 NMR spectrometer with a CryoProbeProdigy BB ATM probe head operating at 400.25 MHz (^{1}H) or a Bruker AVIII HD 500 NMR spectrometer with a CryoProbeProdigy ATM probe head and operating at 500.13 (^{1}H) and 130.35 MHz (^{27}Al). Chemical shifts are reported in δ values in ppm relative to external Me_4Si and, if not otherwise stated, referenced using the chemical shift of the solvent ^{2}H lock resonance frequency and Ξ = 25.145020% for ^{13}C, Ξ = 32.083974% for ^{11}B, Ξ = 26.056859 % for ^{27}Al and Ξ = 94.094011 % for ^{19}F.[1] ^{1}H and ^{13}C spectra have been referenced on specific values for the respective solvent signal. The proton and carbon signals were assigned where possible via a detailed analysis of ^{1}H, ^{13}C, ^{1}H-^{1}H COSY, ^{1}H-^{1}H NOESY, ^{1}H-^{13}C HSQC, ^{1}H-^{13}C HMBC NMR spectra.

Young-type teflon-valve borosilicate NMR tubes have been used throughout the study.

Mass spectrometry

Mass spectra were recorded by the Zentrale Analytik within the Faculty of Chemistry, Göttingen applying a Liquid Injection Field Desorption Ionisation-technique on a JEOL accuTOF instrument with an inert-sample application setup under argon atmosphere. The injection capillary was washed several times with dry, distilled and inertly injected toluene before the samples were injected. Samples usually had a concentration of 1 – 2 mmol/L in toluene and were prepared in a glovebox.

Starting materials and reagents

1-(3',5'-Bis-(trifluoromethyl)phenyl)-2,3,4,5-tetrakis(3',5'-di-*tert.*-butylphenyl)-borole was prepared as previously reported.[2]

Cp^*SiMe_3,[3] Cp^*AlBr_2,[4] $(Cp^*Al)_4$[4] and Cp*Ga[5] were prepared along procedures as reported in the literature. $(AlCp^*)_4$ was recrystallised twice from benzene. GaCp* was distilled and stored at –40°C in a freezer.

Synthesis and Analytical Data

η⁵-[1-(3′,5′-Bis-(trifluoromethyl)phenyl)-2,3,4,5-tetrakis(3‘,5‘-di-*tert.*-butylphenyl)-borole]- η⁵-Cp*-Aluminocene

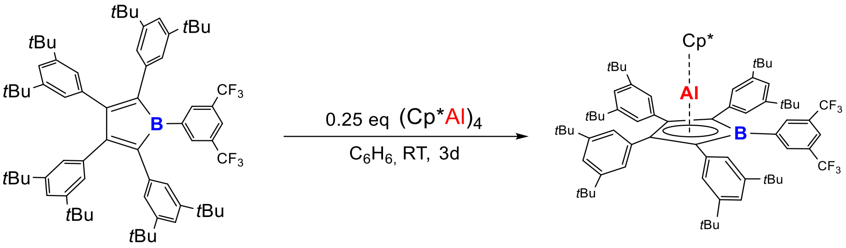

Molecular Weight: 1029,24 | **Molecular Weight: 1191,46**

In a glovebox, to a suspension of $(AlCp^*)_4$ (30 mg, 46.3 µmol, 0.25 eq) in benzene (3 mL), an intensely green solution of 1-(3′,5′-Bis-(trifluoromethyl)phenyl)-2,3,4,5-tetrakis(3‘,5‘-di-*tert.*-butylphenyl)-borole **A** (190.3 mg, 0.185 mmol, 1 eq) in dry, degassed benzene (1 mL) was added at ambient temperature at once and the mixture was stirred for 72h. Over the course of the slow reaction, undissolved $(Cp^*Al)_4$ is continuously dragged into solution and the mixture slowly turns yellow. ^{1}H-NMR spectroscopic examination reveals clean conversion. The solvent is removed under reduced pressure and the pale-yellow residue is dissolved in toluene (ca. 2-3 mL). The solution is stored at –40°C for two days. Fractions of crystalline yield can be collected and drying under vacuum gives a combined yield of η⁵-[1-(3′,5′-Bis-(trifluoromethyl)-phenyl)-2,3,4,5-tetrakis(3‘,5‘-di-*tert.*-butylphenyl)-borole]-η⁵-Cp*-Aluminocene (**1**) (180 mg, 0.151 mmol, 82%) of a colourless to pale yellow crystalline material. Crystals grow from saturated benzene solutions at ambient temperature or toluene or hexane solutions at –40°C.

NMR: ^{1}**H** (400.25 MHz, 298.2K, C_6D_6, C_6D_5H at 7.15 ppm): 7.93 (br s, 2H, *o-H*$_{ar1}$), 7.79 (br s, 1H, *p-H*$_{ar1}$), 7.29 (t, $^{4}J_{HH}$ = 1.8 Hz, 2H, *p-H*$_{ar2,5}$), 7.25 (t, $^{4}J_{HH}$ = 1.8 Hz, 2H, *p-H*$_{ar3,4}$), 6.97 (br s, 4H, *o-H*$_{ar2,5}$), 6.89 (br s, 4H, *o-H*$_{ar3,4}$), 2.16 (s, 15H, Cp*-*Me*), 1.19 (s, 36H, Ar$_{2,5}$-C(*Me*)$_3$), 1.16 (s, 36H, Ar$_{3,4}$-C(*Me*)$_3$).

^{13}**C{^{1}H}** (100.61 MHz, 300K, C_6D_6, solvent signal at 128.0 ppm):149.8 (*m-C*$_{ar2,5}$), 149.7 (*m-C*$_{ar3,4}$), 144.2 (broad, *ipso- C*$_{ar1}$), 138.5 (*ipso-C*$_{ar2,5}$*)*, 135.9 (*ipso-C*$_{ar3,4}$), 135.7 (br q, J_{CF} = 3 Hz, *o-C*$_{ar1}$), 130.1 (q, J_{CF} = 32 Hz, *m-C*$_{ar1}$), 128.4 (s, putatively borole-*C*$_{3,4}$), 126.6 (*o-C*$_{ar3,4}$), 126.1 (superimposed with quartet at 124.8 ppm, *o-C*$_{ar2,5}$), 124.8 (q, J_{CF} = 273 Hz, *C*F$_3$), 119.2 (*p-C*$_{ar3,4}$), 119.1 (m, *p-C*$_{ar1}$), 118.5 (*p-C*$_{ar2,5}$), 118.0 (br s, putatively borole-*C*$_{2,5}$), 117.7 (Cp*-*C*Me), 34.8 (Ar$_{2,5}$-*C*(Me$_3$)), 34.7 (Ar$_{3,4}$-*C*(Me$_3$)), 31.58 (Ar$_{3,4}$-C(*Me*$_3$)), 31.55 (Ar$_{2,5}$-C(*Me*$_3$)), 11.5 (Cp*-C*Me*).

^{11}**B** (128.38 MHz, 298.3K, C_6D_6): 24.6 (broad, $\omega_{1/2}$ = ca. 1050 Hz); (128.38 MHz, 198.2 K, toluene-*d*$_8$): 17.3 (broad, $\omega_{1/2}$ = ca. 500 Hz).

^{19}**F{^{1}H}** (376.45 MHz, 298.4K, C_6D_6): –62.45.

^{27}**Al** (130.35 MHz, 298.2K, C_6D_6): –86.2 (broad, $\omega_{1/2}$ = ca. 2650 Hz).

Elemental Analysis: $C_{78}H_{102}BF_9Al$ calcd C 78.63, H 8.63; observed C 78.68, H 8.63.

LIFDI-MS: calcd exact mass: 1190.78 m/z; observed m/z: 1189.7 (20%), 1190.7 (100%), 1191.7 (80%), 1192.7 (30%), 1193.7 (10%).

Crystallographic details

Crystals grow from concentrated benzene solutions at ambient temperature or toluene or hexane solutions at –40°C. Numerous attempts to change the crystallisation conditions have been made.

Crystals from benzene, toluene and hexane have been examined on a diffractometer. All crystals did not reveal reflections at higher resolutions than ca. 1.2 Å. Closer examination of the structures revealed that the crystals tend to be twinned and systematically reveal in all cases a disorder of the whole molecule which accounts for the poor resolutions. The data clearly allow the identification of the key structural fragment of an aluminium atom that is located in the center between the borole- and Cp-rings and thus confirms the η^5-coordination mode. Modelling of the severe disorder lead to a rather poor data-to-parameter ratio.

Opposed to ambient atmosphere the crystals suspended in oil rapidly lose any crystallinity and crystal examination and picking was only efficiently possible using an XTEMP-setup.

For further detail of the refinement and modelling of the structure please see below (Crystallographic Section).

Crystal crop from benzene:

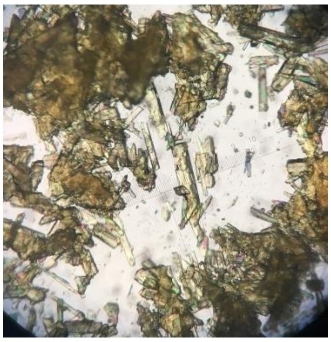

Crystal crop from toluene:

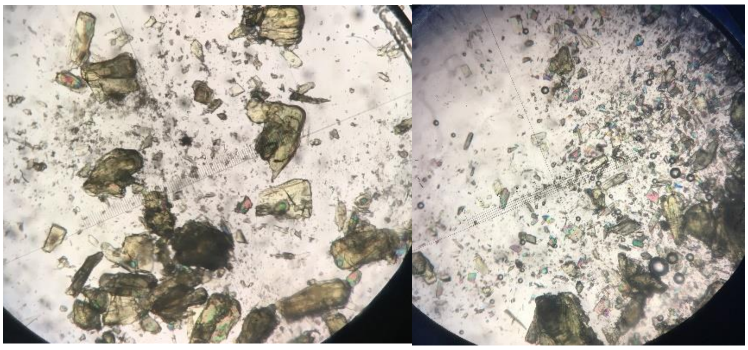

Tabulated crystallographic data for **1**.

	**1 from Toluene**	**1 from Benzene**	**1 from Hexane**
CCDC number	1935771	not deposited	not deposited
empirical formula	$C_{78}H_{102}AlBF_6$ + solvent	$C_{78}H_{102}AlBF_6$ + solvent	$C_{78}H_{102}AlBF_6$ + solvent
formula weight	1191.38	1191.38	1191.38
T / K	100(2)	100(2)	100(2)
λ / Å	0.71073	0.71073	0.71073
crystal system	monoclinic	monoclinic	monoclinic
space group	$P2_1/n$	$P2_1/n$	$P2_1/n$
a / Å	14.770(4)	14.763(2)	14.665(2)
b / Å	17.075(5)	7.123(2)	16.908(2)
c / Å	34.408(10)	33.817(3)	33.643(3)
β / °	97.27(2)	97.82(2)	102.37(2)
V / $Å^3$	8608(4)	8469(2)	8148(2)
Z	4	4	4
ρ / Mg m^{-3}	0.919	0.934	0.971
μ / mm^{-1}	0.070	0.071	0.074
F(000)	2568	2568	2568
crystal size / mm^3	0.415 x 0.309 x 0.100	0.416 x 0.121 x 0.100	0.305 x 0.143 x 0.061
θ range / °	1.193 to 18.122	1.216 to 15.897	1.239 to 18.012
index ranges	$-12 \leq h \leq 12$	$-11 \leq h \leq 11$	$-8 \leq h \leq 12$
	$-14 \leq k \leq 14$	$0 \leq k \leq 13$	$-14 \leq k \leq 14$
	$-29 \leq l \leq 30$	$0 \leq l \leq 26$	$-29 \leq l \leq 29$
refl. Collected	75486	63300	13076
indep. reflections/ R_{int}	5992 / 0.0692	4052 / 0.0458	5609 / 0.0419
completeness to $θ_{max}$	99.4 %	99.7 %	99.8 %
data/restraints/parameters	5992 / 8425 / 1276	4052 / 8469 / 1276	5609 / 8645 / 1276
GooF	1.044	1.077	1.355
final R indices [I>2sigma(I)] R_1 / wR_2	0.0830 / 0.2047	0.0959 / 0.2246	0.1096 / 0.3132
R indices (all data) R_1 / wR_2	0.1024 / 0.2193	0.1023 / 0.2283	0.1520 /0.3463
largest diff. peak and hole / e$Å^{-3}$	0.399 / -0.264	0.241 / -0.188	0.470 and -0.421
absorption correction	multiscan	multiscan	multiscan
twin fractions	-	0.918 / 0.082	-
SQUEEZE: V_{solv} / $Å^3$	1856	1734	1374
SQUEEZE: V_{solv} / V_{cell}	22 %	20 %	17 %
SQUEEZE: number of e^-	544	466	312

Plots of the NMR spectra

1H-NMR-spectrum of 1-[3',5'-(CF3)2(C6H3)]-2,3,4,5-(3',5'-tBu2(C6H3)-borole x AlCp* in C6D6
C6D5H at 7.15 ppm

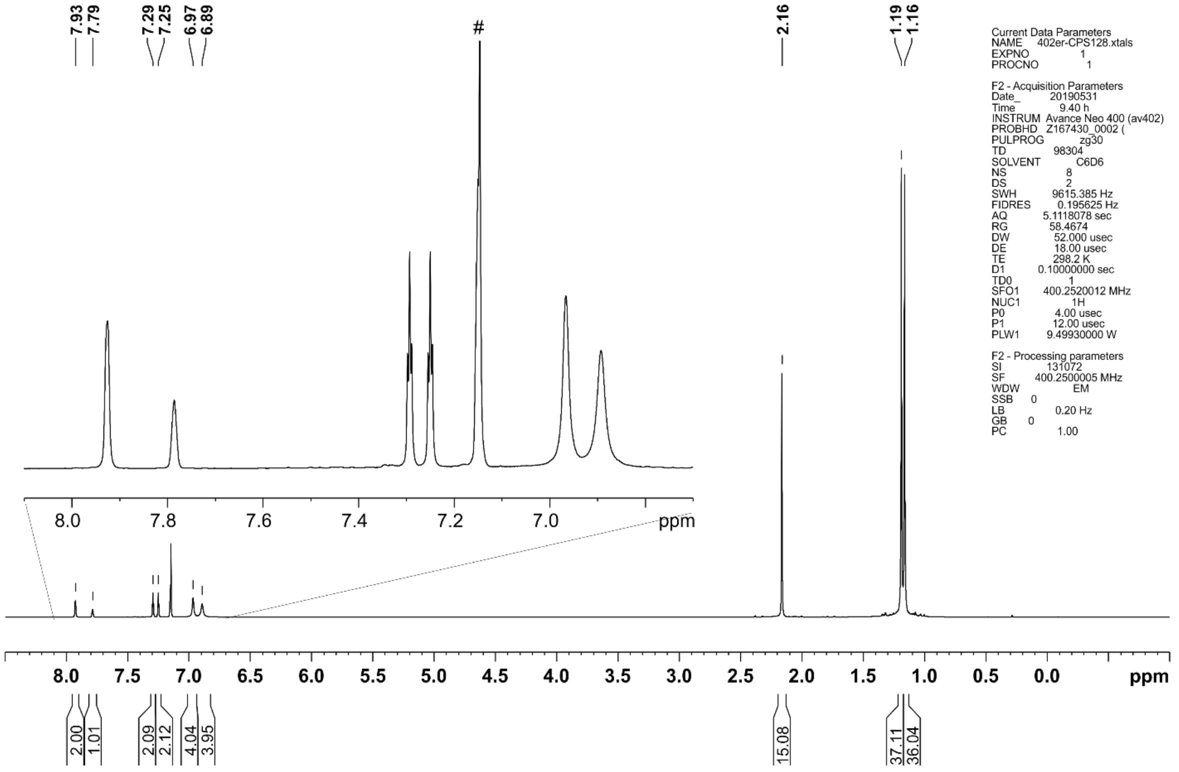

1H-NMR-spectrum of
1-[3',5'-(CF3)2(C6H3)]-2,3,4,5-[3',5'-tBu2(C6H3)]-borole x AlCp*
in tol-d8 at -75°C
referenced to (C6D5)CD2H at 2.08 ppm

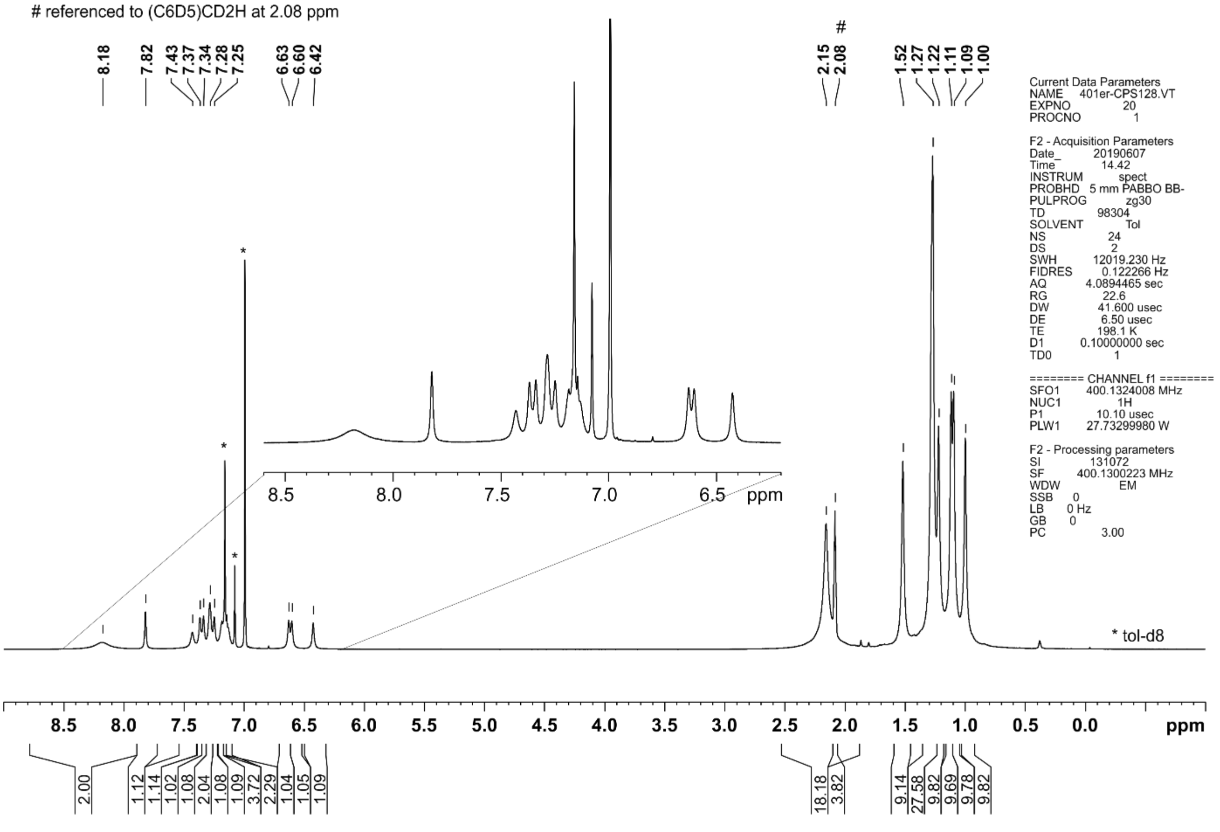

13C{1H}-NMR-spectrum of 1-[3',5'-(CF3)2(C6H3)]-2,3,4,5-[3',5'-tBu2(C6H3)]-borole x AlCp* in C6D6
referenced to C6D6 at 128.0 ppm

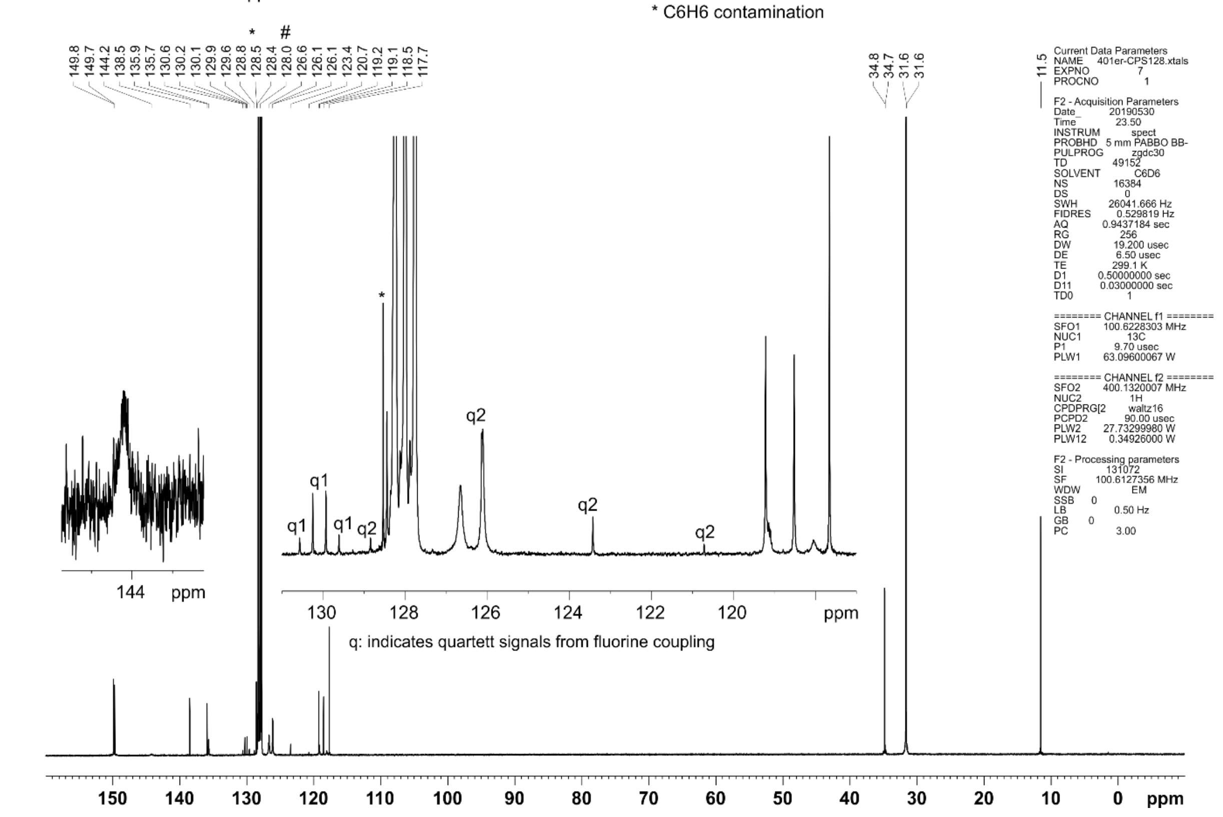

19F{1H}-NMR-spectrum of 1-[3',5'-(CF3)2(C6H3)]-2,3,4,5-[3',5'-tBu2(C6H3)]-borole x AlCp* in C6D6

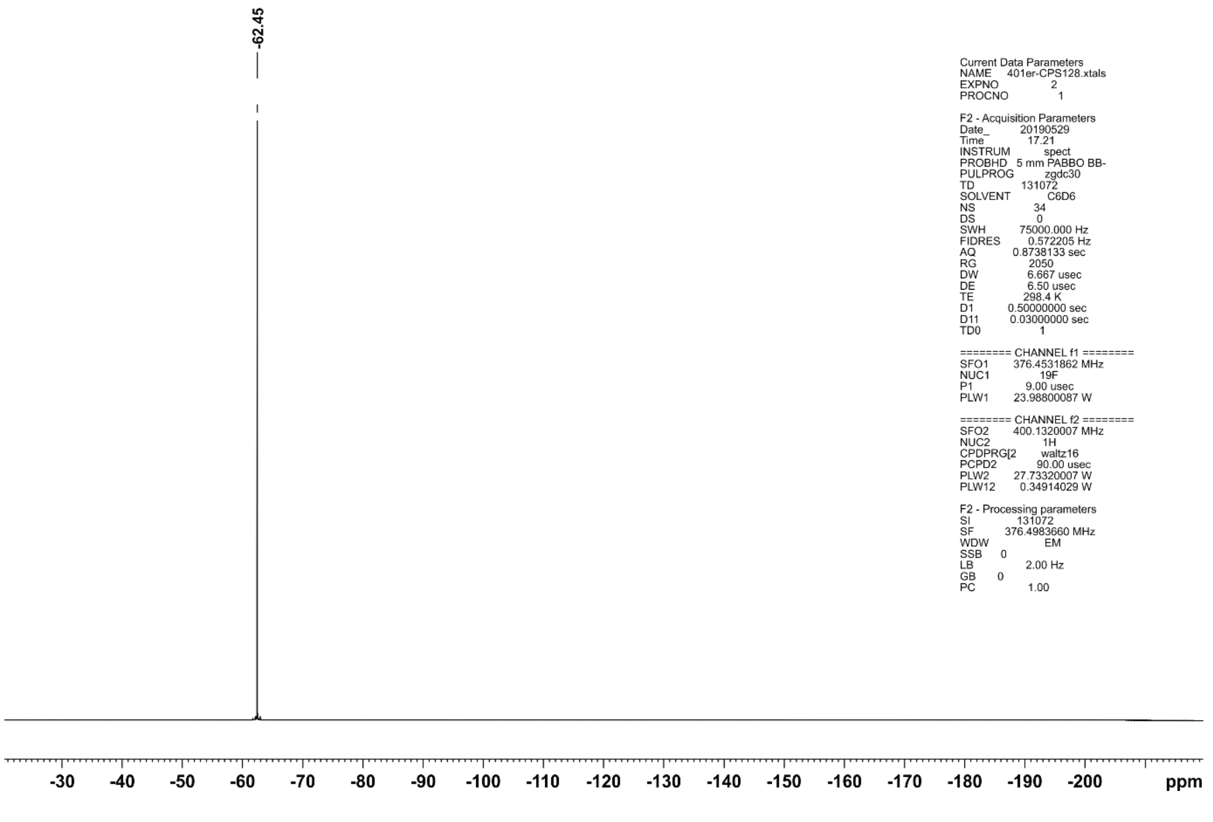

11B-NMR spectrum (background suppressed) of 1-[3',5'-(CF3)2(C6H3)]-2,3,4,5-[3',5'-tBu2(C6H3)]-borole x AlCp* in C6D6
(standard borosilicate nmr tube)

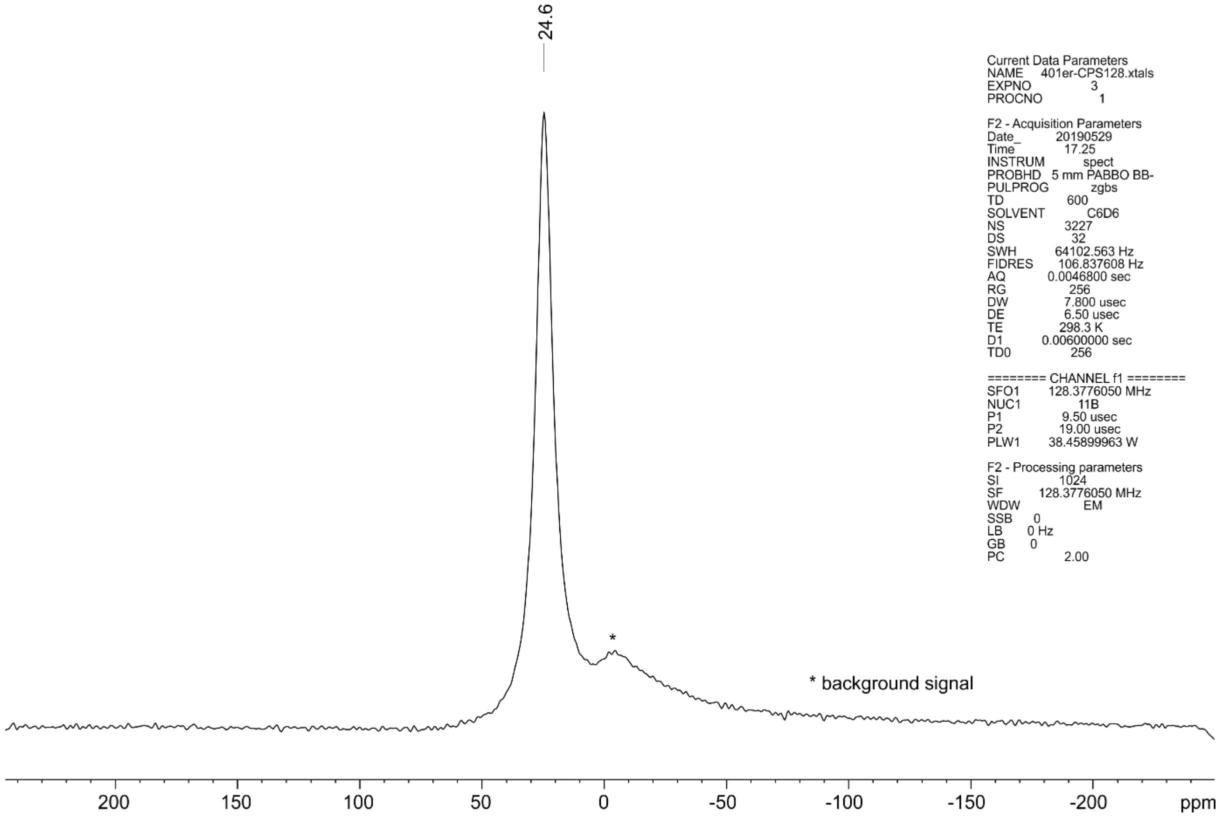

11B-NMR spectrum (background suppressed) of
1-[3',5'-(CF3)2(C6H3)]-2,3,4,5-[3',5'-tBu2(C6H3)]-borole x AlCp*
in toluene-d8 at -75°C
(standard borosilicate nmr tube)

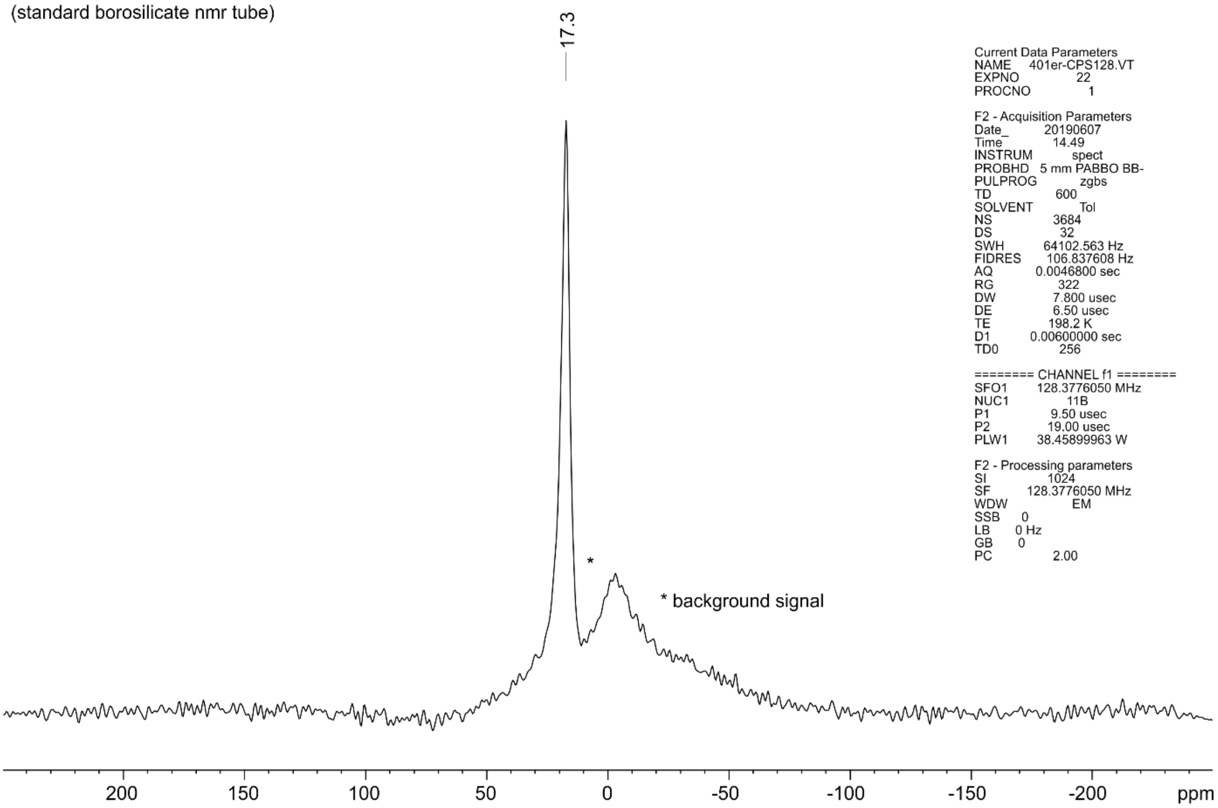

27Al NMR spectrum of 1-[3',5'-(CF3)2(C6H3)]-2,3,4,5-[3',5'-tBu2(C6H3)]-borole x GaCp* in C6D6

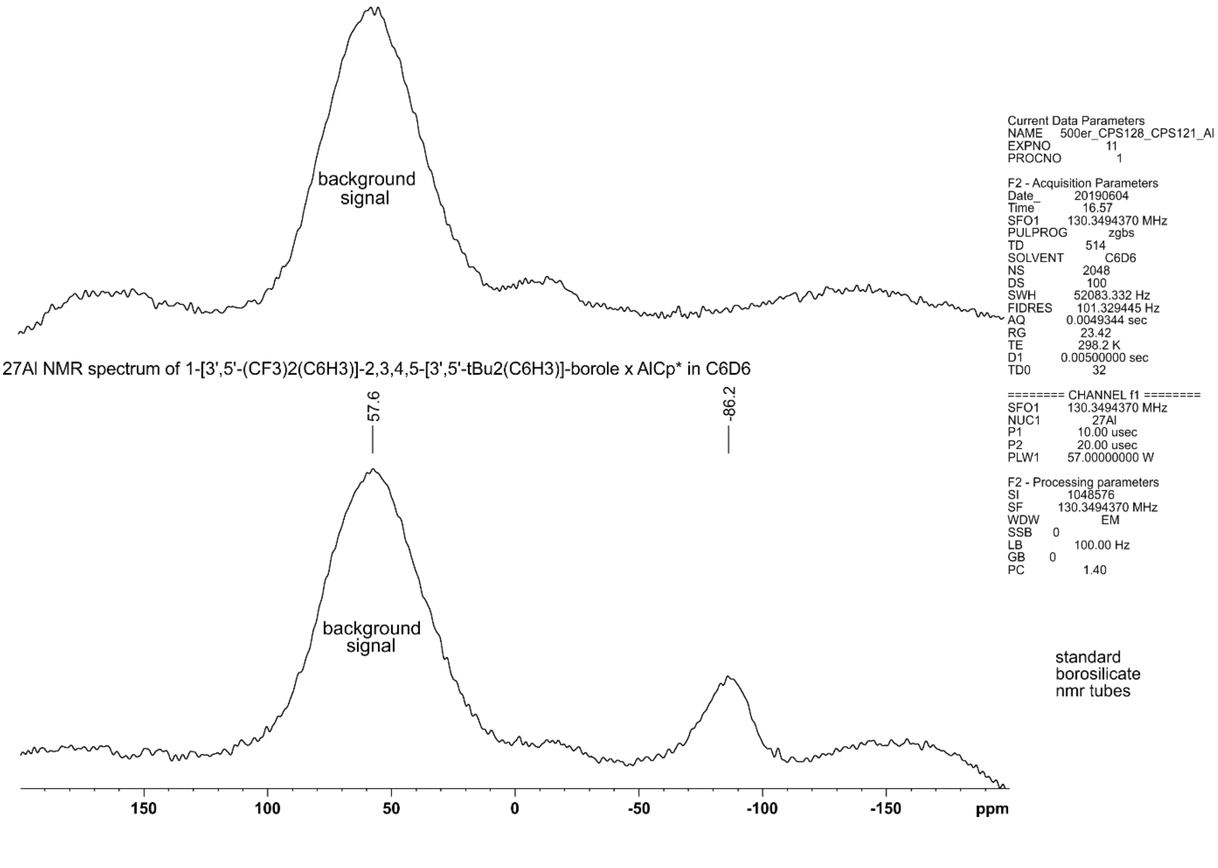

VT-^{1}H-NMR stackplot of Aluminium-complex 1 in toluene-d_8. Referenced to toluene-d_8 at 2.08 ppm.

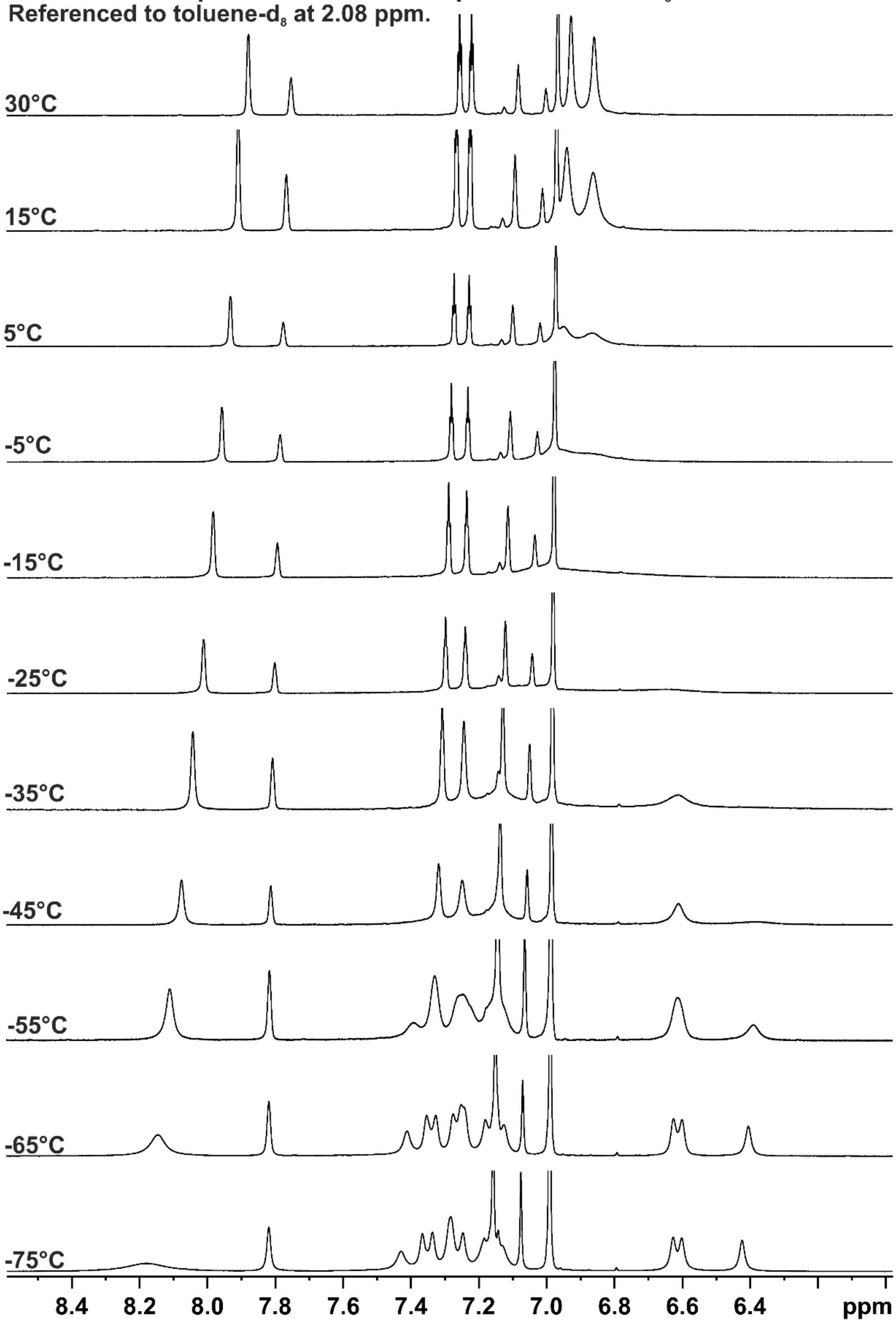

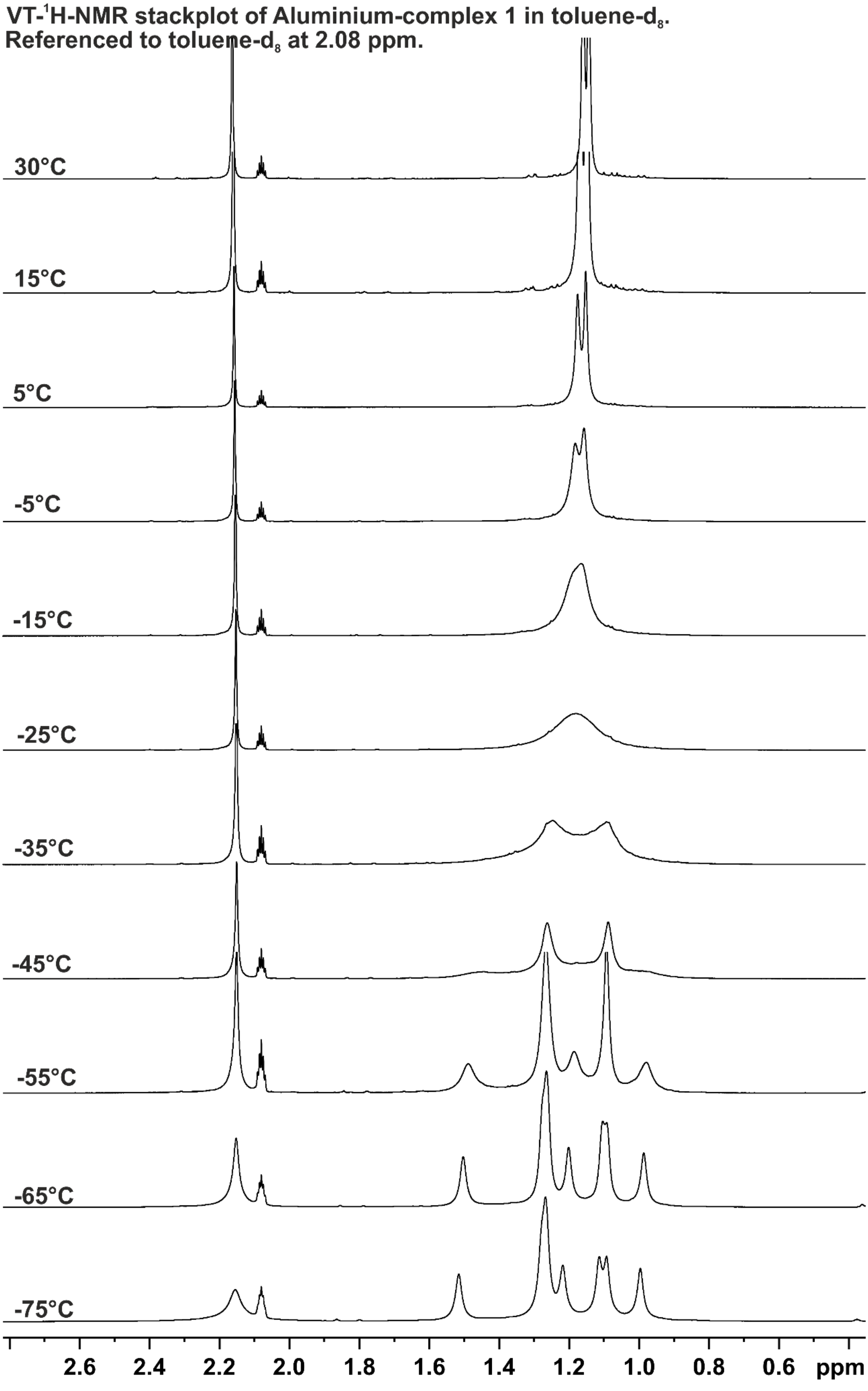
VT-^{1}H-NMR stackplot of Aluminium-complex 1 in toluene-d_8.
Referenced to toluene-d_8 at 2.08 ppm.
30°C
15°C
5°C
-5°C
-15°C
-25°C
-35°C
-45°C
-55°C
-65°C
-75°C
2.6
2.4
2.2
2.0
1.8
1.6
1.4
1.2
1.0
0.8
0.6
ppm

Plots of the LIFDI-MS spectra

Acq. Data Name: csindli00021-1
Creation Parameters: Average(MS[1] Time:0.84..0.85)
External Sample Id: CPS128

Ionization Mode: FD+

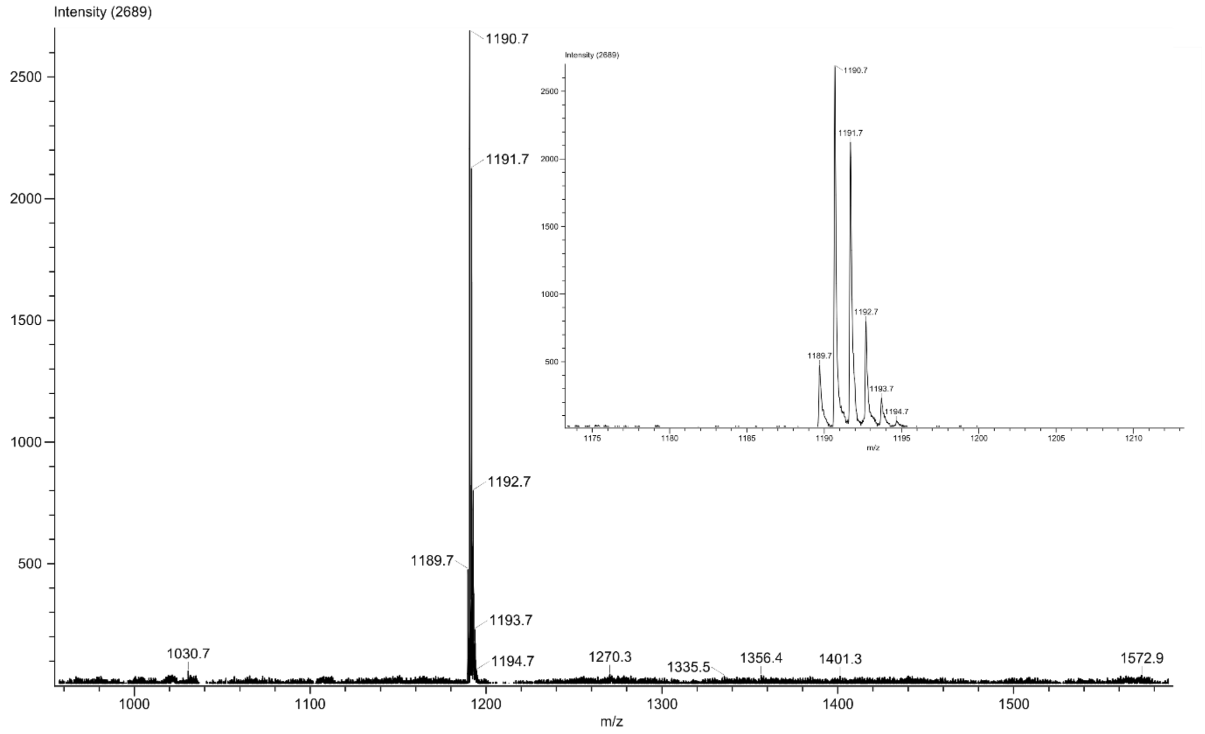

η¹-[1-(3',5'-Bis-(trifluoromethyl)phenyl)-2,3,4,5-tetrakis(3',5'-di-*tert.*-butylphenyl)-borole]- η⁵-Cp*-Gallium

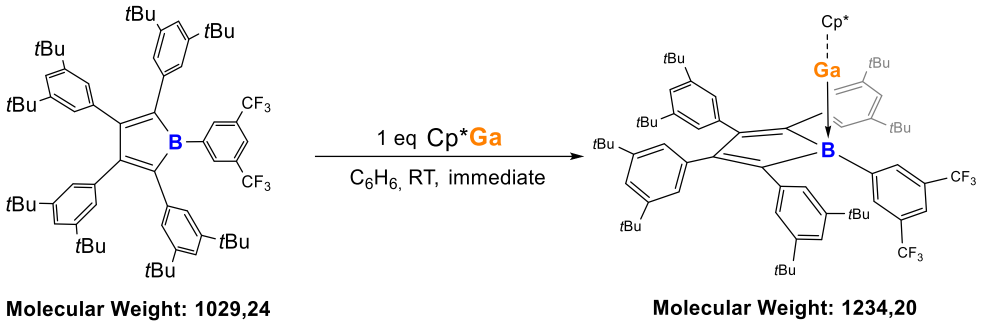

In a glovebox, to an intensely green solution of 1-(3',5'-Bis-(trifluoromethyl)phenyl)-2,3,4,5-tetrakis(3',5'-di-*tert.*-butylphenyl)-borole **A** (158.2 mg, 0.154 mmol, 1 eq) in dry, degassed benzene (2 mL) was added a solution of GaCp* (31.5 mg, 0.153 mmol, 1 eq) in benzene (1 mL) at ambient temperature at once and the mixture immediately changed from green to a bright orange-red. ^{1}H-NMR spectroscopic examination reveals clean conversion. The solvent is removed under reduced pressure and the orange residue is dried for several hours to give the product **2** in virtually quantitative yield (188 mg, 0.152 mmol, 99%) as an orange solid. The compound is very soluble in hydrocarbons. Crystals were grown by concentrating benzene solutions through slow evaporation of the solvent at ambient temperature. Small fractions of crystalline material can also be obtained from storage of very concentrate solutions in pentane at –40°C.

NMR:

^{1}**H** (400.13 MHz, 298.2K, C_6D_6, C_6D_5H at 7.15 ppm): 7.86 (br s, 2H, *o-H$_{ar1}$*), 7.64 (br s, 1H, *p-H$_{ar1}$*), 7.23 (t, $^{4}J_{HH}$ = 1.9 Hz, 2H, *p-H$_{ar3,4}$*), 7.17 (t, $^{4}J_{HH}$ = 1.8 Hz, 2H, *p-H$_{ar2,5}$*), 7.11 (d, $^{4}J_{HH}$ = 1.8 Hz, 4H, *o-H$_{ar2,5}$*), 6.97 (d, $^{4}J_{HH}$ = 1.9 Hz, 4H, *o-H$_{ar3,4}$*), 1.89 (s, 15H, Cp*-*Me*), 1.17 (s, 36H, Ar$_{3,4}$-C(*Me*)$_3$), 1.12 (s, 36H, Ar$_{2,5}$-C(*Me*)$_3$).

^{13}**C{^{1}H}** (100.62 MHz, 298.7K, C_6D_6, solvent signal at 128.0 ppm): 151.2 (borole C$_{3,4}$), 150.7 (broad, *ipso*-C$_{ar1}$), 150.2 (*m-C$_{ar3,4}$*), 149.7 (*m-C$_{ar2,5}$*), 149.6 (borole C$_{2,5}$), 140.1 (*ipso-C$_{ar2,5}$*), 140.0 (*ipso-C$_{ar3,4}$*), 135.8 (br q, *o-C$_{ar1}$*), 130.4 (q, J_{CF} = 32 Hz, *m-C$_{ar1}$*), 125.1(*o-C$_{ar3,4}$*), 124.6 (*o-C$_{ar2,5}$*), 124.5 (q, J_{CF} = 273 Hz, *C*F$_3$), 119.4 (m, *p-C$_{ar1}$*), 119.34 (*p-C$_{ar3,4}$*), 119.29 (*p-C$_{ar2,5}$*), 114.3 (Cp*-*C*Me), 34.7 (Ar$_{3,4}$-*C*(Me$_3$)), 34.6 (Ar$_{2,5}$-*C*(Me$_3$)), 31.7 (Ar$_{3,4}$-C(*Me$_3$*)), 31.5 (Ar$_{2,5}$-C(*Me$_3$*)), 9.6 (Cp*-C*Me*).

^{11}**B** (128.38 MHz, 298.2K, C_6D_6): 7.3 (broad, $\omega_{1/2}$ = ca. 1550 Hz); (128.38 MHz, 223.1 K, toluene-*d*$_8$): –0.4 (broad; due to superimposition with background no meaningful linewidth assignment possible).

^{19}**F{^{1}H}** (376.45 MHz, 298.3 K, C_6D_6): –62.46.

Elemental Analysis: $C_{78}H_{102}BF_9Ga$ calcd C 75.91, H 8.33; observed C 75.60, H 8.50.

LIFDI-MS: calcd exact mass: 1232.72 m/z; observed only m/z patterns of the free borole (minor) and [borole × H_2O] (major).

Crystallographic details

Crystals suitable for X-ray analysis grow from benzene solutions carefully concentrated at ambient temperature by evaporation and storage of the very concentrated liquid for a few days.

Opposed to ambient atmosphere the crystals suspended in oil rapidly lose colour and crystallinity, and crystal examination and picking was performed using an XTEMP-setup.

Crystal crop from benzene:

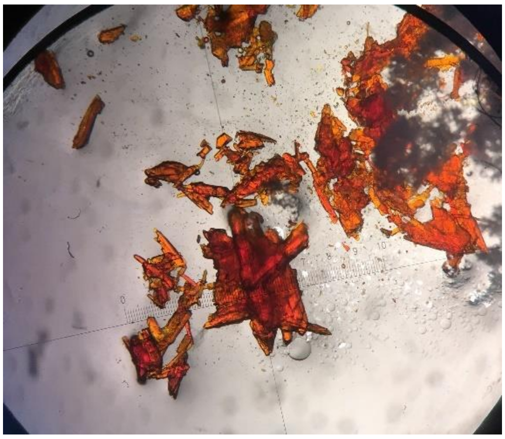

Tabulated crystallographic data for **2**.

Compound	**2**
CCDC number	1935772
Empirical formula	$C_{78}H_{102}BGaF_6$× (C_6H_6)
Formula weight	1312.23
T [K]	100(2)
Λ [Å]	0.71073 (Mo, Kα)
Crystal system	orthorhombic
Space group	*Pbca*
a [Å]	17.4388(13))
b [Å]	29.760(2)
c [Å]	29.925(3)
α [°]	90
β [°]	90
γ [°]	90
V [Å^3]	15531(2)
Z	8
ρ [Mg m^{-3}]	1.122
μ [mm^{-1}]	0.41
F(000)	5616
Crystal size [mm^3]	0.36 × 0.13 × 0.11
Theta range [°]	1.4 – 27.9
Index ranges	–21≤ h ≤ 22
	–30 ≤ k ≤ 39
	–35 ≤ l ≤ 39
Refl. collected	76338
Indep. refl. / [R(int)]	18542/ 0.058
Completeness to θ_{max}	99.8%
Data/restraints/parameter	18542/1003/912
GooF	1.01
Final *R* indices [*I* >2sigma(*I*)] *R*1 / *wR*2	0.045 / 0.114
R indices (all data) *R*1 / *wR*2	0.080/ 0.099
Largest diff. peak & hole [eA^{-3}]	0.52/ –0.36
Absorption correction	multiscan

Plots of the NMR spectra

1H-NMR-spectrum of 1-[3',5'-(CF3)2(C6H3)]-2,3,4,5-(3',5'-tBu2(C6H3))-borole x GaCp* in C6D6
referenced to C6D5H at 7.15 ppm

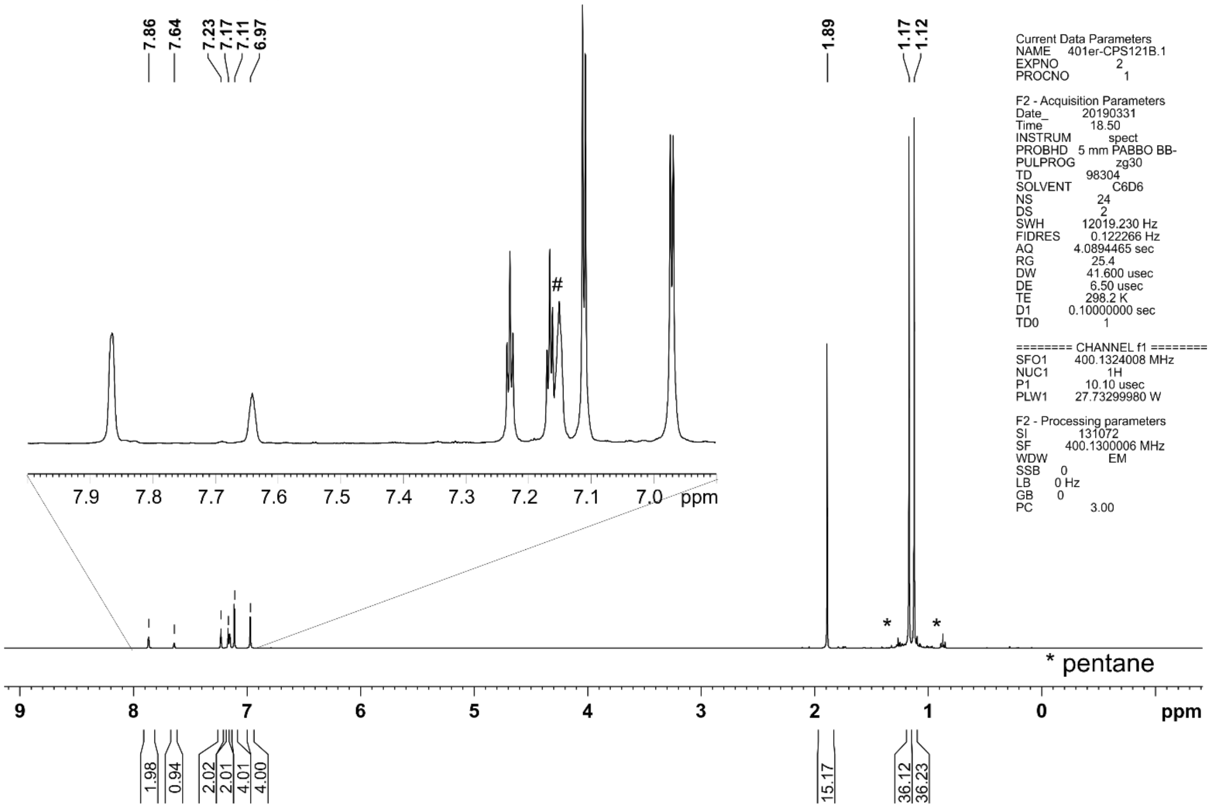

1H-NMR-spectrum of 1-[3',5'-(CF3)2(C6H3)]-2,3,4,5-[3',5'-tBu2(C6H3)]-borole x 1.1 eq GaCp*
referenced to (C6D5)CD2H at 2.08 ppm

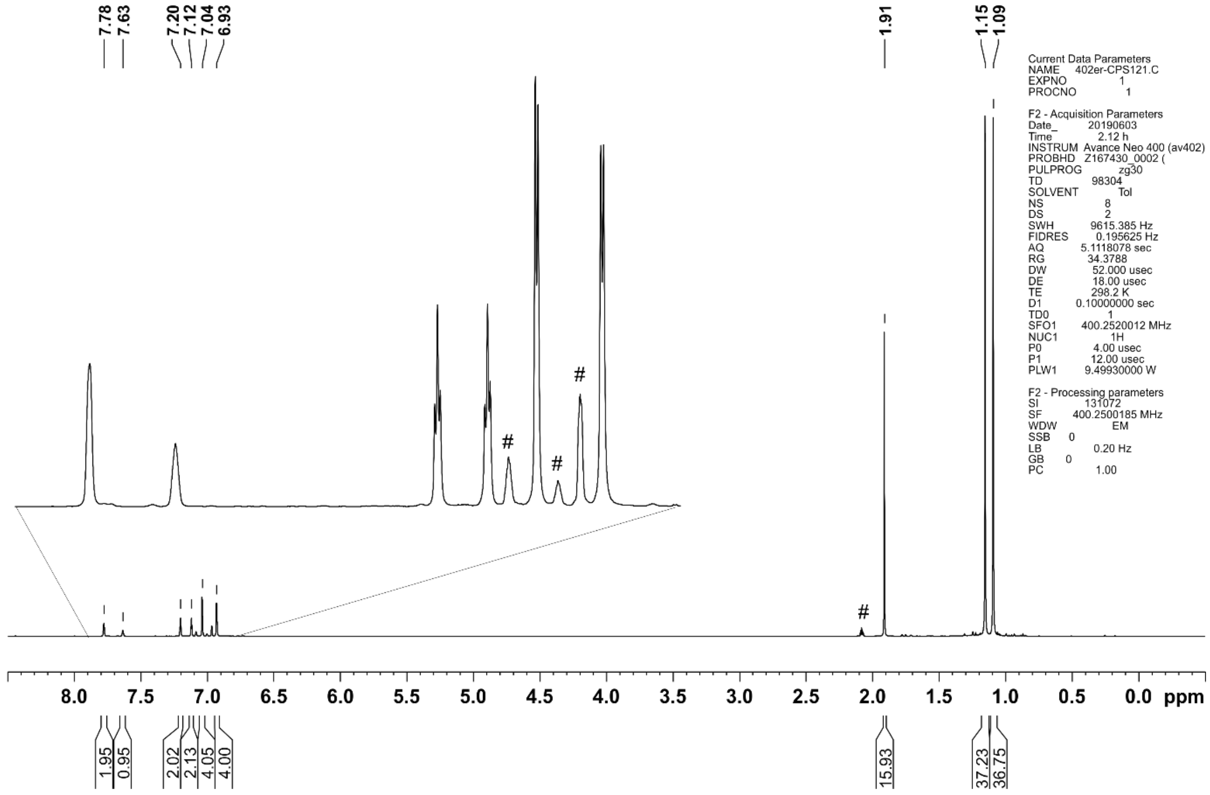

1H-NMR-spectrum of 1-[3',5'-(CF3)2(C6H3)]-2,3,4,5-[3',5'-tBu2(C6H3)]-borole x GaCp* in toluene-d8
at -50°C, # referenced to (C6D5)CD2H at 2.08 ppm

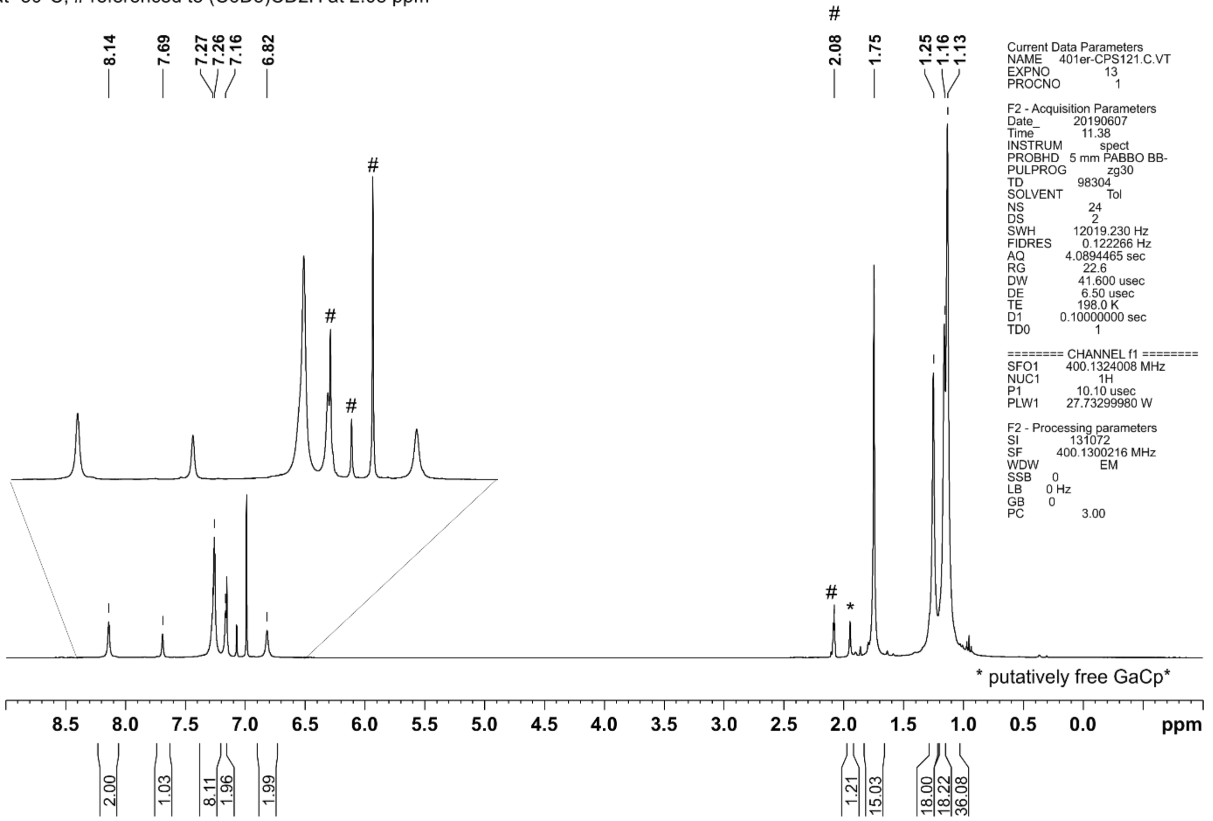

13C{1H}-NMR-spectrum of 1-[3',5'-(CF3)2(C6H3)]-2,3,4,5-[3',5'-tBu2(C6H3)]-borole x GaCp* in C6D6
referenced to C6D6 at 128.0 ppm

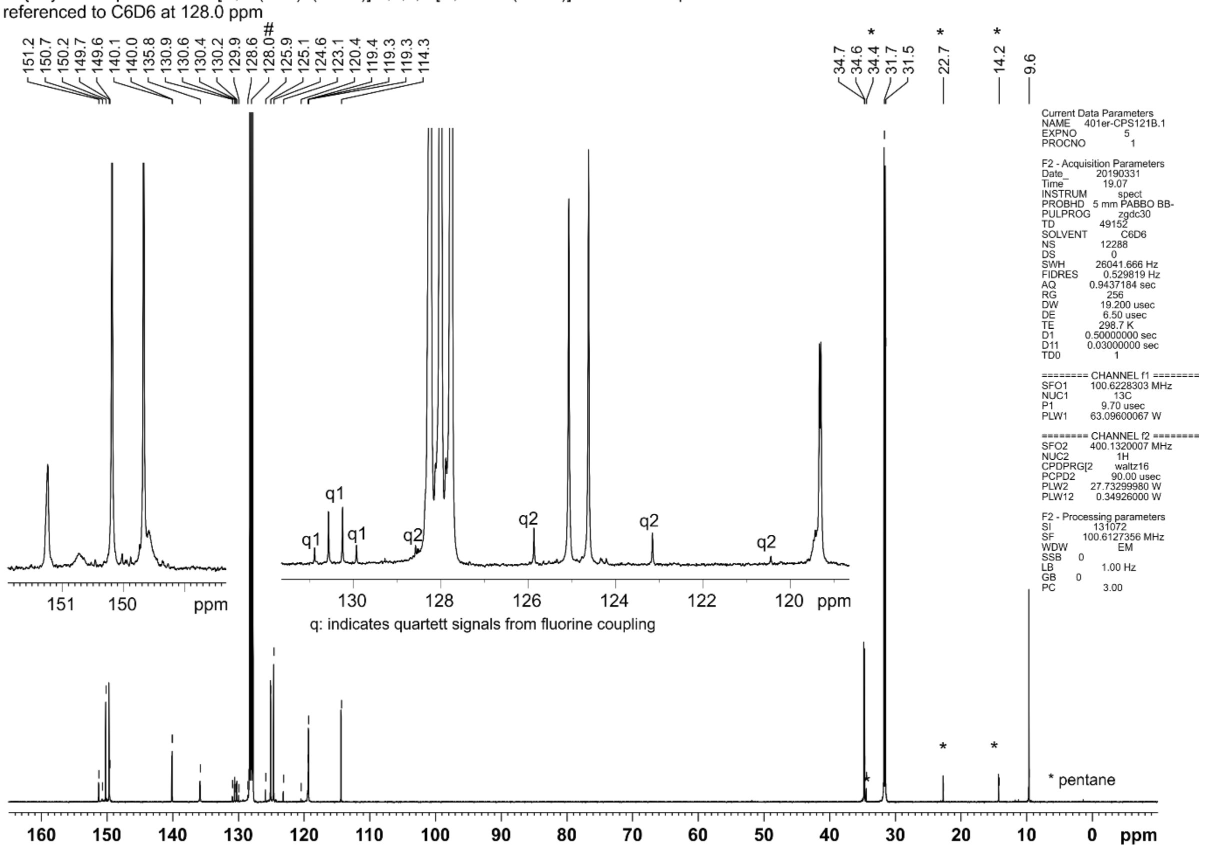

19F{1H}-NMR-spectrum of 1-[3',5'-(CF3)2(C6H3)]-2,3,4,5-(3',5'-tBu2(C6H3))-borole x GaCp* in C6D6

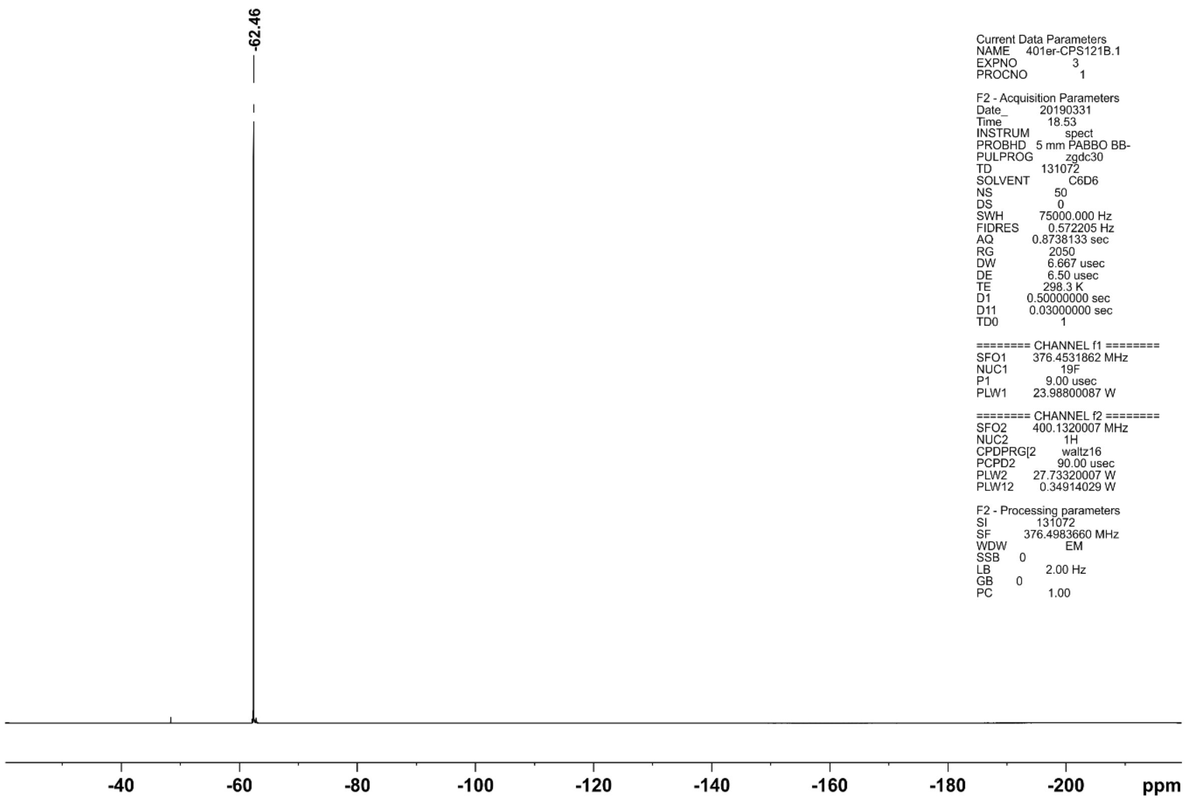

11B-NMR spectrum (background suppressed) of 1-[3',5'-(CF3)2(C6H3)]-2,3,4,5-(3',5'-tBu2(C6H3))-borole x GaCp* in C6D6

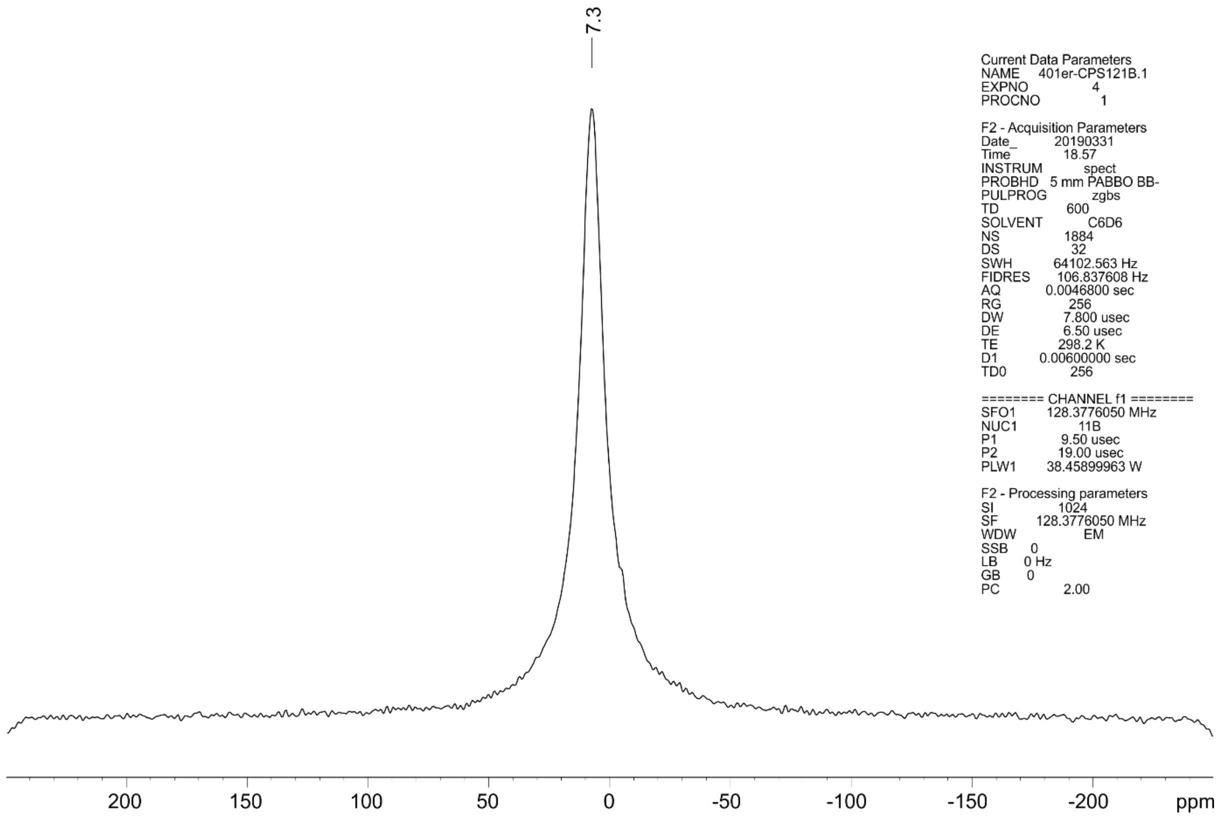

11B-NMR spectrum (background suppressed) of 1-[3',5'-(CF3)2(C6H3)]-2,3,4,5-[3',5'-tBu2(C6H3)]-borole x GaCp*
in toluene-d8 at -50°C

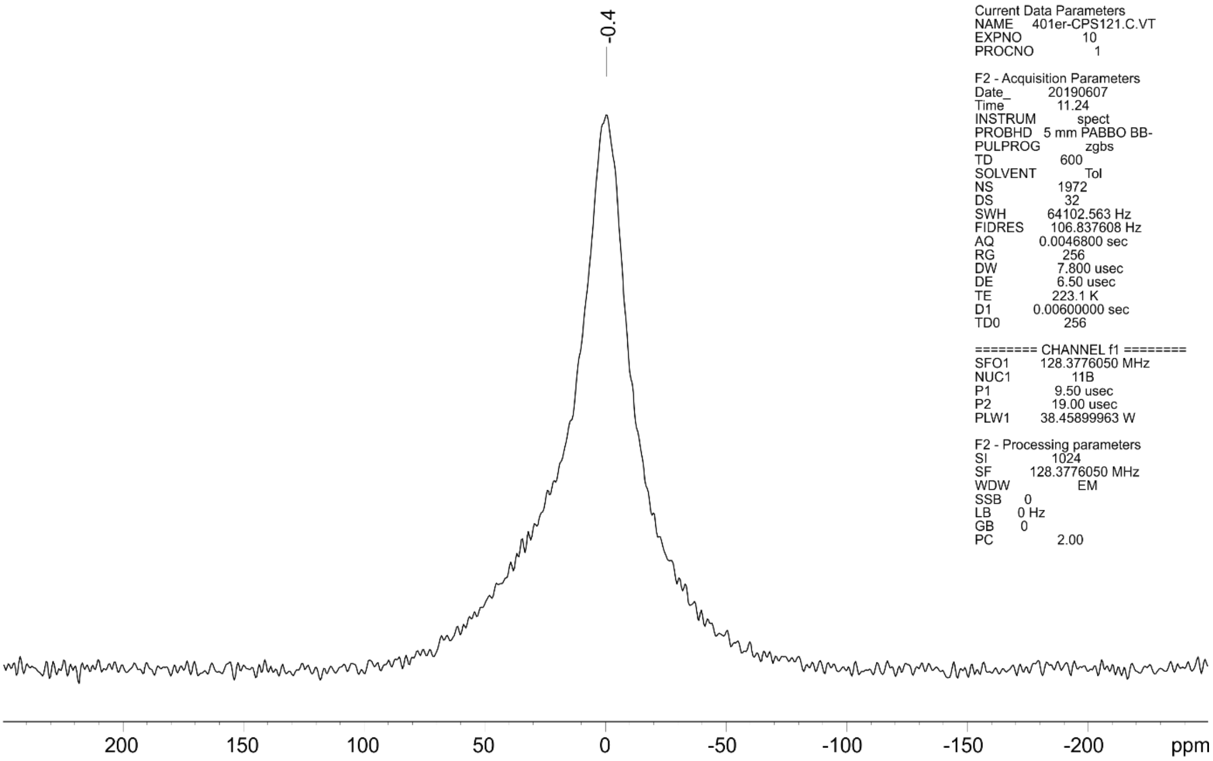

VT-^{1}H-NMR stackplot of Gallium-complex 2 in toluene-d_8.
Referenced to toluene-d_8 at 2.08 ppm.

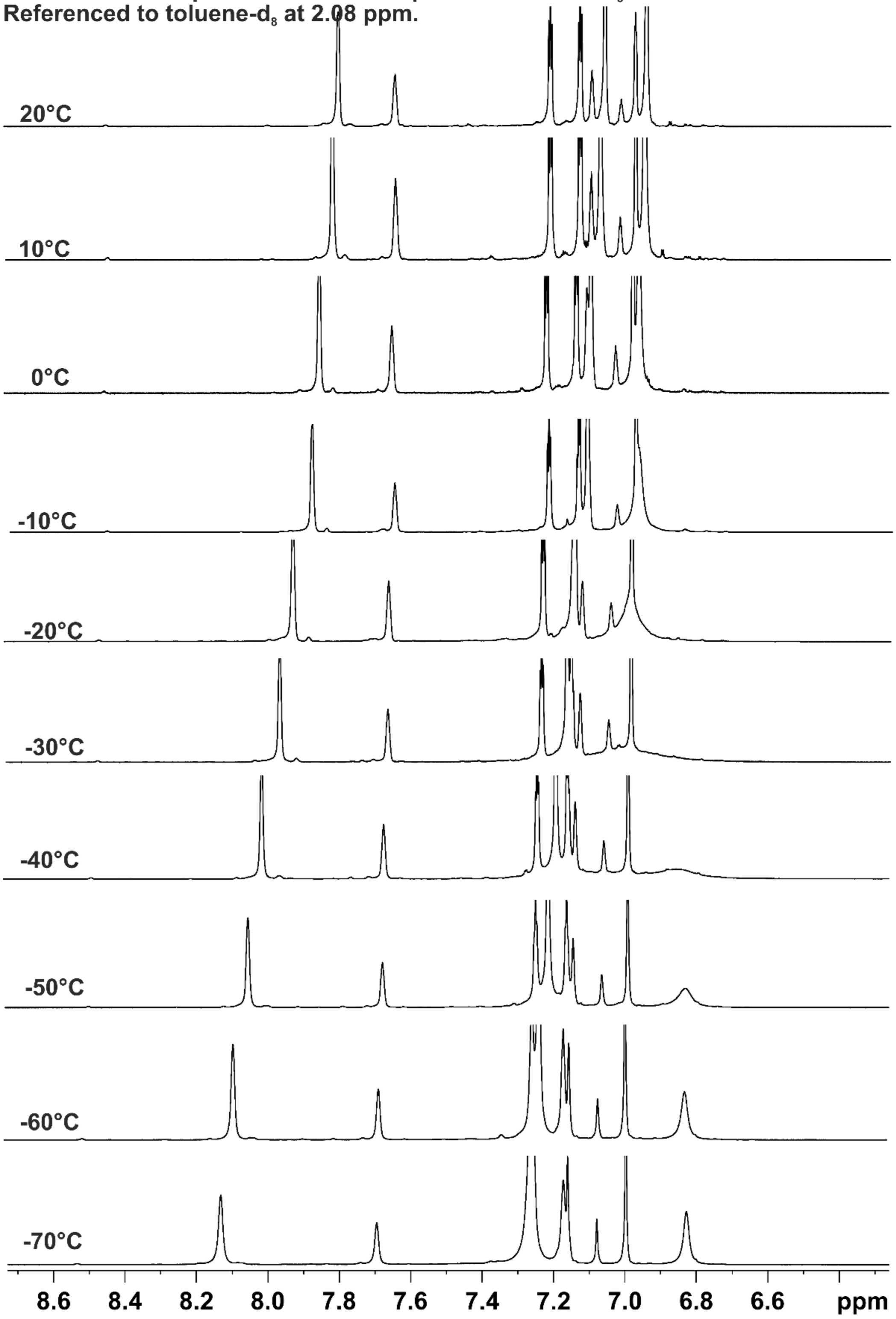

VT-^{1}H-NMR stackplot of Gallium-complex 2 in toluene-d_8.
Referenced to toluene-d_8 at 2.08 ppm.

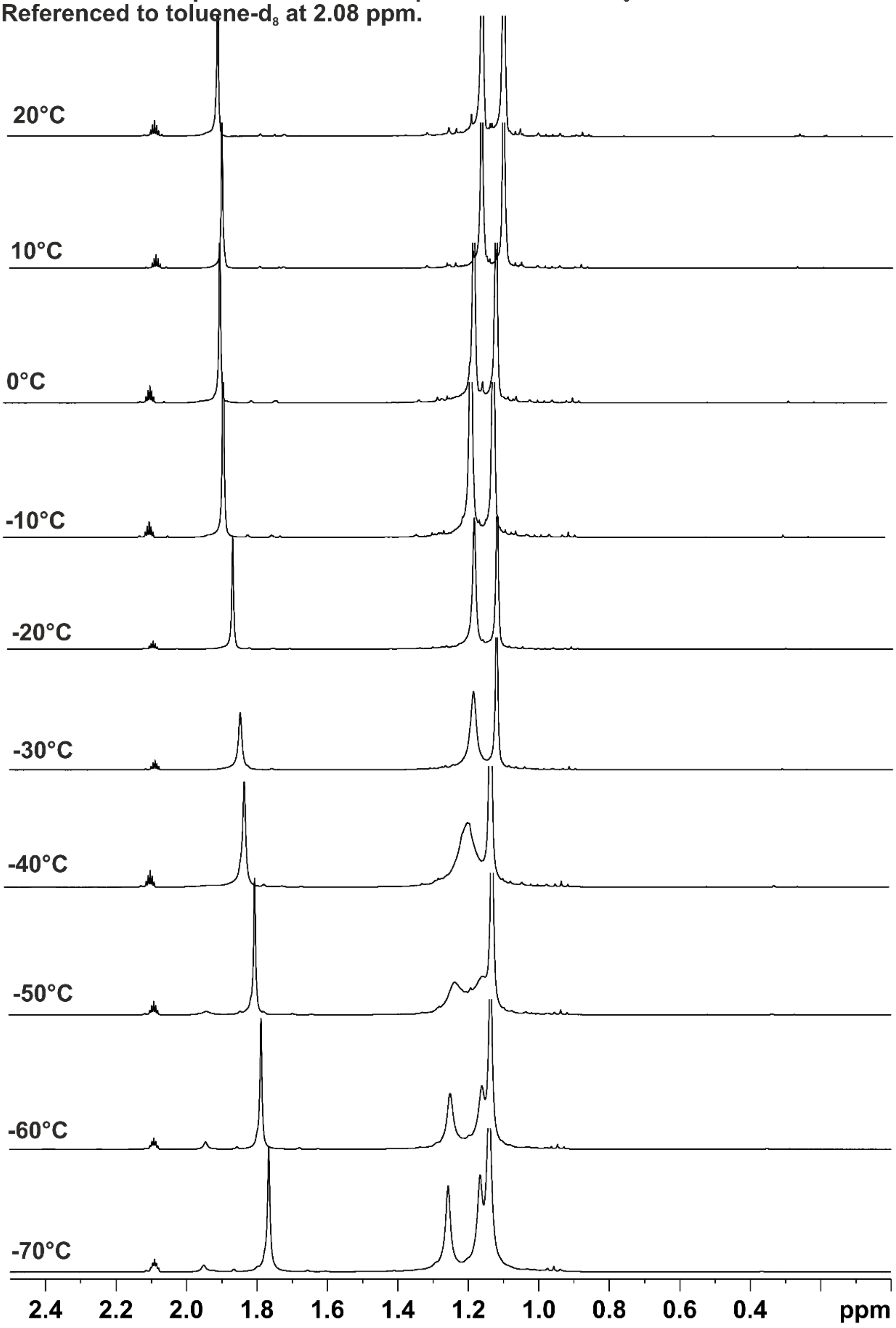

Plots of the LIFDI-MS spectra

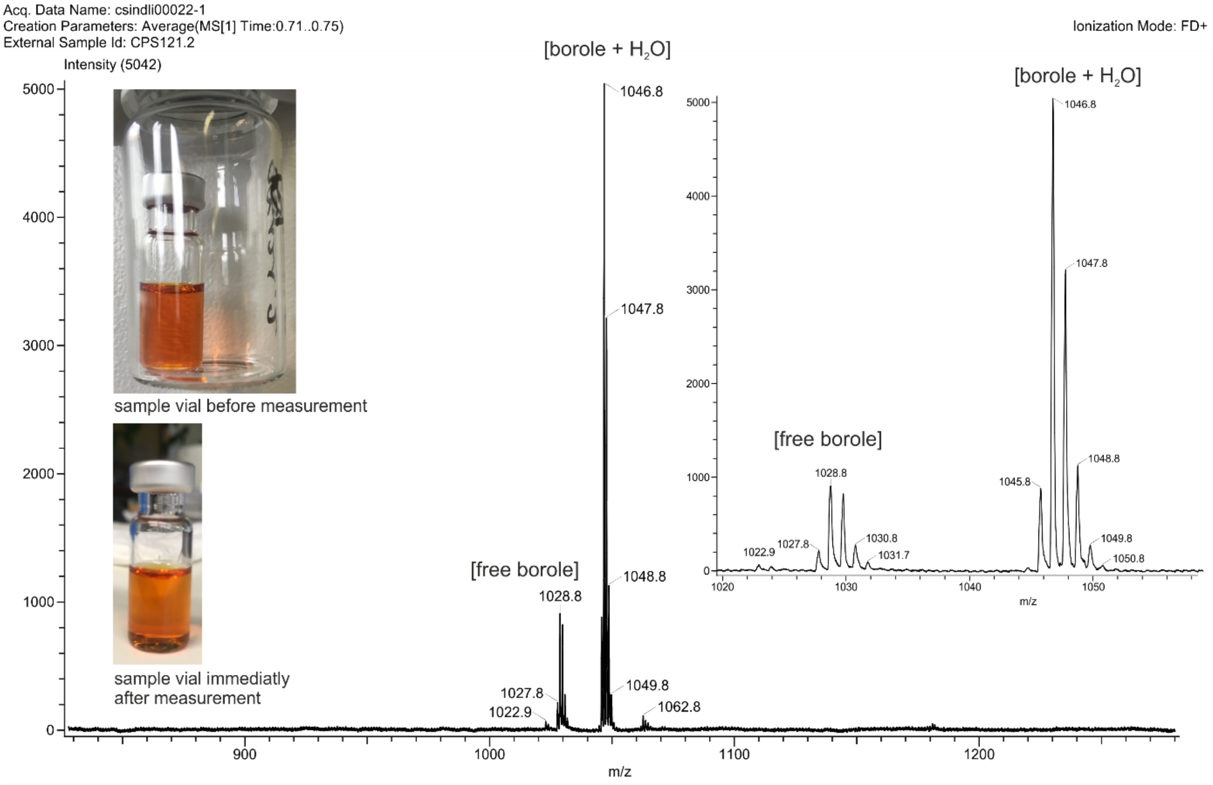

No signals (e.g. GaCp* derivatives) were detected in the lower mass region 0 - 800 m/z

Control Experiment (Free Borole in Toluene)

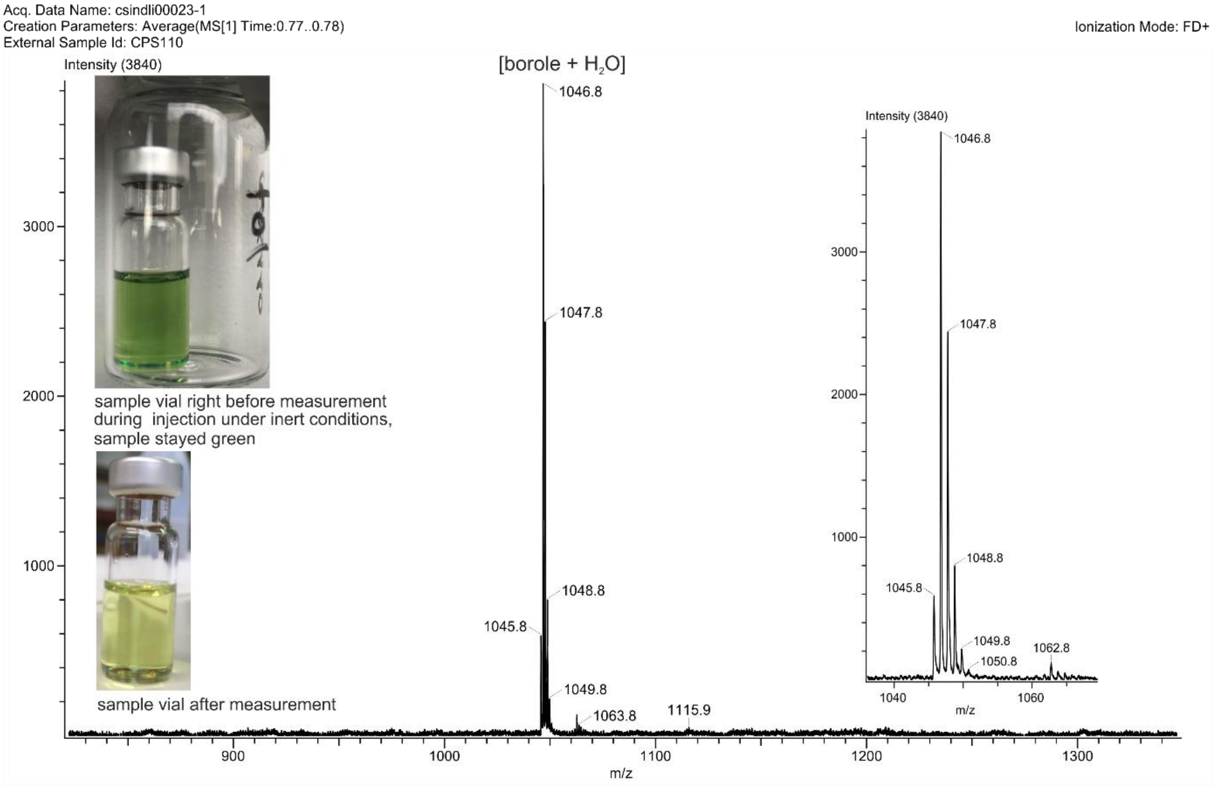

Crystallographic Details

Data Acquisition and Processing

X-ray data for **1,** and **2** were collected on Bruker APEX II CCD diffractometers with either Mo Kα radiation from a IµS or spinning anode source. The data were integrated using SAINT implemented in Brukers APEX3 programme suite.[6] SADABS [7] or TWINABS were used for multi-scan absorption correction.[8] Structure solution was performed with SHELXT[9] and refined using SHELXL[10] along the graphical user interphase of ShelXle.[11] In some cases DSR has been applied to treat disordered solvent molecules.[12] All hydrogen atoms were placed with a riding model. Further details on the individual data sets are tabulated in the analytical section of each compound. All structures were deposited with the CCSD.

Crystallographic and Refinement Details 1

Crystals of compound **1** were obtained from three different solvents (toluene, benzene and hexane) from concentrate solutions at ambient or low temperature (–40°C). The crystals are stable under argon atmosphere but lose their crystallinity under ambient conditions in inert oil within minutes. Crystals were therefore mounted with an XTEMP 2 device.

As the crystals of **1** from benzene were twinned, the two reciprocal lattices were sorted using RLATT from within the Bruker Apex 3 2018.7-2 GUI. All three datasets were integrated using SAINT 8.38A.

All three structures showed disorder within the solvent molecules in solvent accessible voids and within the majority (the entire borole sub unit) of the structure itself, which in consequence results in very poor intensity of reflections with a resolution higher than about 1.2 Å. To fit the solvent molecules with as little parameters as possible, the solvent molecules within the moieties were fitted using the SQUEEZE model, as implemented in PLATON.[13]

The disorder of the Ph* and Xyl^F groups were treated differently. The Ph* moiety was modelled using a modified mesityl group as included in the DSR programme with all the non *tBu*-methyl group positions being refined as a rigid-body.[12] The positions of the bound methyl groups were refined freely (see figure on the right).

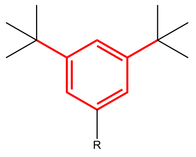

Representation of the used rigid group. The positions of the red atoms were refined as rigid group, the black methyl groups were refined freely.

Within the five membered borole unit, C_α and C_β positions were restrained to have similar 1,2 and 1,3 distances. The resulting target symmetry of the restrains would be equivalent to a mirror plane through the boron atom and the opposing carbon-carbon bond. All C-C distances from the borole ring to the outer substituents were refined to be equivalent as well. All *tert*-butyl groups were restrained to have similar 1,2 and 1,3 distances. Equivalent restraints were applied to the trifluoromethyl groups.

Atomic displacement parameters of atoms within the disordered borole moiety were refined to be have similar U_{ij} components to their neighbours (SIMU). Additionally, rigid body restraints for the atomic displacement parameters were applied to these atoms (RIGU).

With the very similar electron density pattern of a (C-Ph*) vs a (B-Xyl^F) moiety, the quasi five-fold symmetry, as well as the disorder, pose the question, whether there are additional orientations. All putative combinations of boron positions for the two disorders were evaluated, with the reported structures showing a significantly lower *R*-value than the alternatives. The difference can, in large part, be attributed to the fit of the CF_3 groups. The model should therefore represent the two main positions of the borole moiety. However, due to the nature of the disorder and the limited resolution, additional minor occupation where the ring overlaps but is rotated differently, cannot be ruled out.

Despite the considerable efforts the resulting data-to-parameter ratio was still low for all three structures. This is an inherent result from the structure itself, as already mentioned before. However, the derived features are similar between all three structures and consistent with all other experimental and especially theoretical results.

Tabulated values for the key structural features of the "Aluminocene" **1** from various data sets. Please note, that there are differences of the Al–B distances in Disorder 1 and Disorder 2. This may indicate that the exact assignment/modelling of (C-Ph*) vs.(B-Xyl^F)-units may be incomplete.

	**1 from Toluene**		**1 from Benzene**		**1 from Hexane**	
	Disorder 1	Disorder 2	Disorder 1	Disorder 2	Disorder 1	Disorder 2
**occupation / %**	49.0(2)	51.0(2)	59.4(3)	40.6(3)	63.7(2)	36.3(2)
**d(Al - B) / Å**	2.31(2)	2.13(2)	2.24(2)	2.08(6)	2.25(2)	2.15(2)
**d(Al - $C_{\alpha,1}$) / Å**	2.25(2)	2.00(2)	2.20(2)	2.01(3)	2.12(2)	2.25(2)
**d(Al - $C_{\alpha,2}$) / Å**	2.10(2)	2.22(2)	2.11(3)	2.27(4(	2.23(2)	2.02(2)
**d(Al - $C_{\beta,1}$) / Å**	2.19/2)	2.17(2)	2.15(3)	2.20(5)	2.18(2)	2.30(2)
**d(Al - $C_{\beta,2}$) / Å**	2.12(2)	2.32(2)	2.12(2)	2.35(3)	2.20(2)	2.15(2)
**d(B - $C_{\alpha,1}$) / Å**	1.54(2)	1.53(2)	1.54(2)	1.55(4)	1.54(2)	1.53(2)
**d(B - $C_{\alpha,2}$) / Å**	1.53(2)	1.54(2)	1.54(2)	1.56(4)	1.55(2)	1.56(2)
**d($C_{\alpha,1}$- $C_{\beta,1}$) / Å**	1.46(2)	1.47(2)	1.45(2)	1.48(3)	1.46(2)	1.46(2)
**d($C_{\alpha,2}$- $C_{\beta,2}$) / Å**	1.47(2)	1.47(2)	1.45(2)	1.48(3)	1.45(2)	1.46(2)
**d($C_{\beta,1}$- $C_{\beta,2}$) / Å**	1.42(2)	1.41(2)	1.42(2)	1.42(4)	1.44(2)	1.43(2)

Depictions of the disordered borole subunit within the molecule **1**. Part 1 (Blue), Part 2 (orange). The second fragment is a borole unit rotated by ca. 36° with an inversion of the paddlewheel tilt of the aryl groups. This major disorder, along with further disorder within the *t*-Bu groups causes the low resolution of the obtainable data.

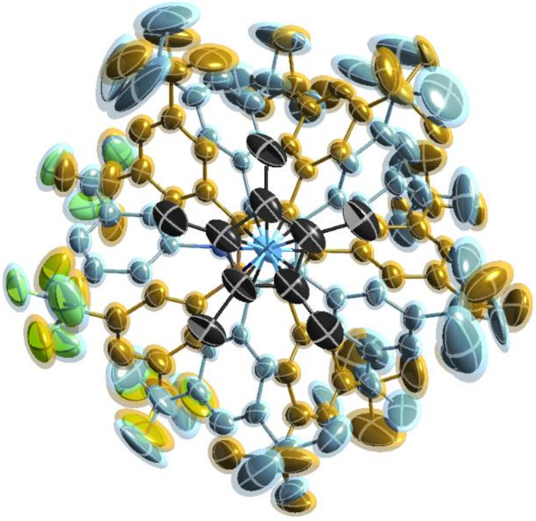

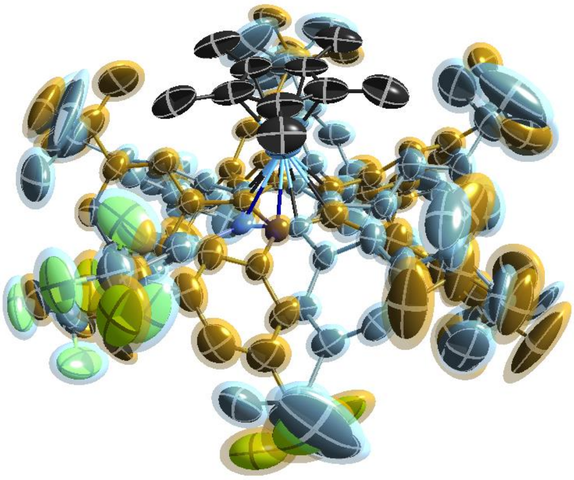

Refinement Details 2

The structure contains one molecule of lattice benzene, which is disordered modelled using SIMU, RIGU and SAME commands. Two *tert.*-butyl groups and a CF_3 group are disordered and each modelled over two positions using SIMU, RIGU and SAME commands.

Computational Details

Structure Optimisation, Frequency Calculation and Thermochemical Approximations

For thermochemical approximations, structures were optimised with Gaussian09.D01[14] applying the BP86 functional[15] and Grimmes D3 dispersion correction[16] with def2-SVP[17] basis sets on all elements. Frequency calculations were performed on these structures and absence of imaginary frequencies confirmed true local minima on the potential energy surface. Thermochemical corrections stem from these calculations. Single point energies were calculated on these structures using a def2-TZVP basis set on all atoms.

Gaussian09 Thermochemistry BP86-D3/def2-TZVP								
	Hartree --->				kcal/mol --->			
	E0	E0+ZVPE[a]	H[a]	G[a]	dE0	dE0+ZVPE	dH*	dG*
AlCp*	-632,722527	-632,508421	-632,493222	-632,54852				
GaCp*	-2315,39782	-2315,18444	-2315,16894	-2315,2255				
**A**	-3268,97933	-3267,62881	-3267,54704	-3267,74717				
**1**	-3901,79893	-3900,23013	-3900,13451	-3900,35853				
**2**	-5584,42566	-5582,85951	-5582,76234	-5582,99303				
**A + AlCp* → 1**	-0,09707216	-0,09290516	-0,09425416	-0,06283816	-60,91	-58,30	-59,15	-39,43
**A + GaCp* → 2**	-0,04850441	-0,04625641	-0,04635541	-0,02036841	-30,44	-29,03	-29,09	-12,78

[a] Thermochemical corrections stem from BP86-D3-def-SVP optimisation and frequency calculations.

Summary GIAO-NMR computations

Computational examination was performed using ORCA (version 4.1.).[18] For numerical accuracy, a gridsize of "5" and a final step gridsize of "6" is applied. GIAO-NMR spectroscopic properties were calculated as implemented as the default in ORCA4.1 applying RIJK-PBE0[19] functional on structures previously optimised using the RI-BP86-D3BJ-def2TZVP/J model chemistry.[15, 17, 20] Input structures were based on X-ray structures of **2** and **A**. For NMR calculations of the reference set of small molecules, def2-TZVPP basis sets were chosen for B, Al and Ga and def2-TZVP for all other elements.

For the rather large molecules **1** and **2**, def2-TZVPP basis sets were chosen for B, Al and Ga, while a def2-TZVP basis was chosen for the core carbon atoms (namely borole C_α and C_β positions, the *ipso*-C_{XylF} atom as well as the inner cyclopentadienyl carbon atoms). A def2-SVP basis set was applied for all other atoms.

The reported ^{11}B-NMR chemical shifts are referenced against the σ_{iso} values obtained for Et_2O-BF_3 with δ_{ref} = 0 ppm.

The reported ^{27}Al-NMR chemical shifts are referenced against the σ_{iso} values obtained for $[Al(H_2O)_6]^{3+}$ with δ_{ref} = 0 ppm.

The reported ^{13}C-NMR chemical shifts are internally referenced against the averaged σ_{iso} values of the five cyclopentadienyl signals with δ_{ref} = [experimentally observed shift in C_6D_6 at RT] ppm

$$\delta_{calc} = \sigma_{ref} - \sigma_{calc}$$

**^{27}Al-NMR**	$\sigma_{iso,calc}$	$\delta_{iso,calc}$	δ_{exp}
$[Al(H_2O)_6]^{3+}$	579.3 (σ_{ref})	0 (ref)	0 (ref)
$[AlCl_4]^-$	458.8	120.5	115.2[21]
$[Cp^*_2Al]^+$	691.8	–112.5	–102.9[21]
tBu_3Al	299.5	279.8	255[22]
**1**	669.3	–90	–86.2

**^{11}B-NMR**	$\sigma_{iso,calc}$	$\delta_{iso,calc}$	δ_{exp}
$BF_3(OEt_2)$	101.2 (σ_{ref})	0 (ref)	0 (ref)
$[Cp^*_2B]^+$	151.7	–50.5	–41.3[23]
Cp^*_2BMe	27.8	73.4	81.9[23]
**1**	82.6	18.6	24.6 (298 K)<br>17.3 (198 K)
**2**	102.1	–0.9	–0.9 (298 K)<br>–0.4 (223 K)

	Compound **1**				Compound **2**			
**^{13}C-NMR**	Cp* *C*Me	C_α	C_β	*ipso*-C_{XylF}	Cp* *C*Me	C_α	C_β	*ipso*-C_{XylF}
$\sigma_{iso,calc}$	63.6<br>181.3[a] (σ_{ref})<br>(62.7, 62.8, 60.3, 66.5, 65.8)	63.4<br>(64.6<br>62.1)	55.2<br>(55.17, 55.29)	36.5	66.8<br>181.1[a] (σ_{ref})<br>(66.8, 65.9, 67.3, 64.6, 69.6)	31.2<br>(32.4, 29.9)	29.4<br>(32.9, 25.8)	29.5
$\delta_{iso,calc}$	117.7 (ref)	117.9	126.1	144.8	114.3 (ref)	149.9	151.7	151.6
δ_{exp}	117.7 (ref)	118.0	128.4	144.2	114.3 (ref)	149.6	151.2	150.7
[a] The external reference ^{13}C $\sigma_{iso,calc}$ value for $SiMe_4$ was calculated to be 186.9 ppm.								

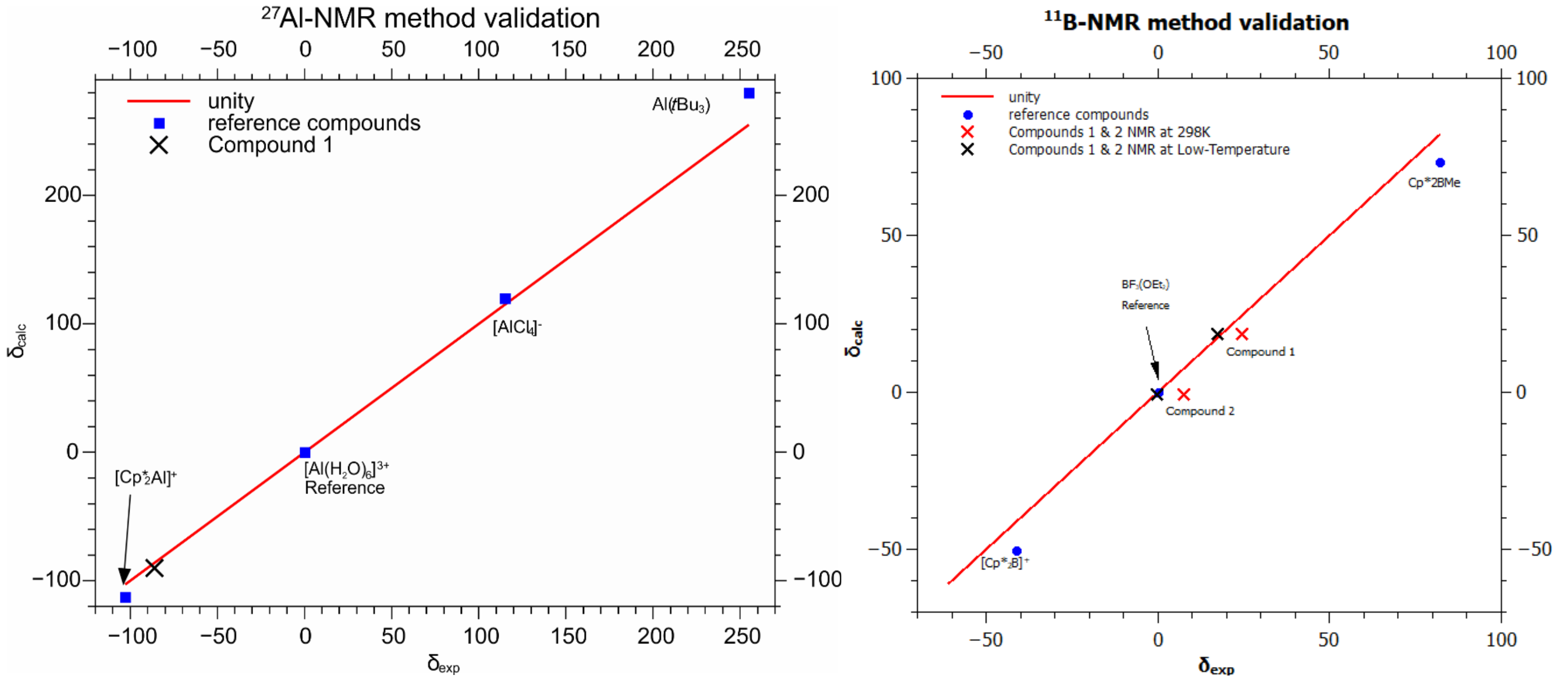

Figure SI Plots of δ_{exp} vs δ_{calc} to allow for an estimation of the reliability of the model chemistry to describe the NMR-chemical shift.

Frontier Orbital Depictions

Selected canonical frontier orbitals from BP86 calculations (*vide* supra) are shown. All drawn at an isosurface value of 0.04 a.u. using the programme ChemCraft for visualisation.[24] All hydrogen atoms are omitted for the sake of clarity.

Compound **1** η^5-[(PhC)$_4$BXyl^F], η^5-Cp*-Al(III)

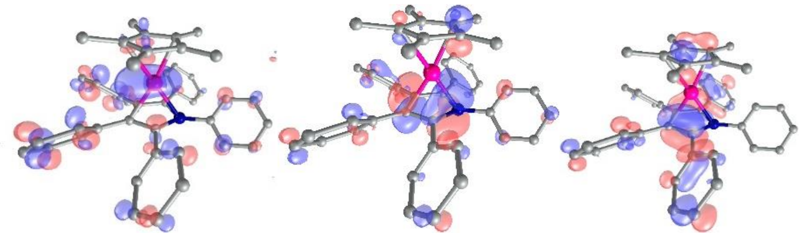

LUMO+2 HOMO HOMO-1

Compound **2** η^1-[(PhC)$_4$BXyl^F], η^5-Cp*-Ga(I)

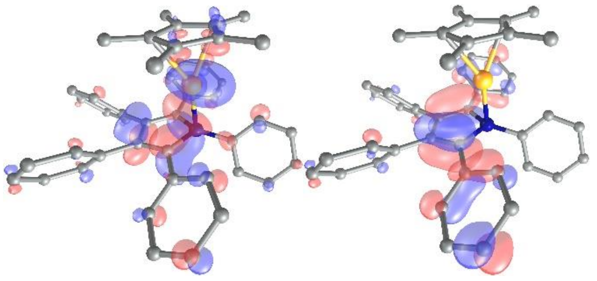

LUMO HOMO

Topology Analyses

Topology analyses and Bader Charge-analyses[25] were carried out using the Multiwfn programme[26] or AIMAll[27] on the RI-BP86-D3BJ-def2TZVP wave function files obtained from ORCA.

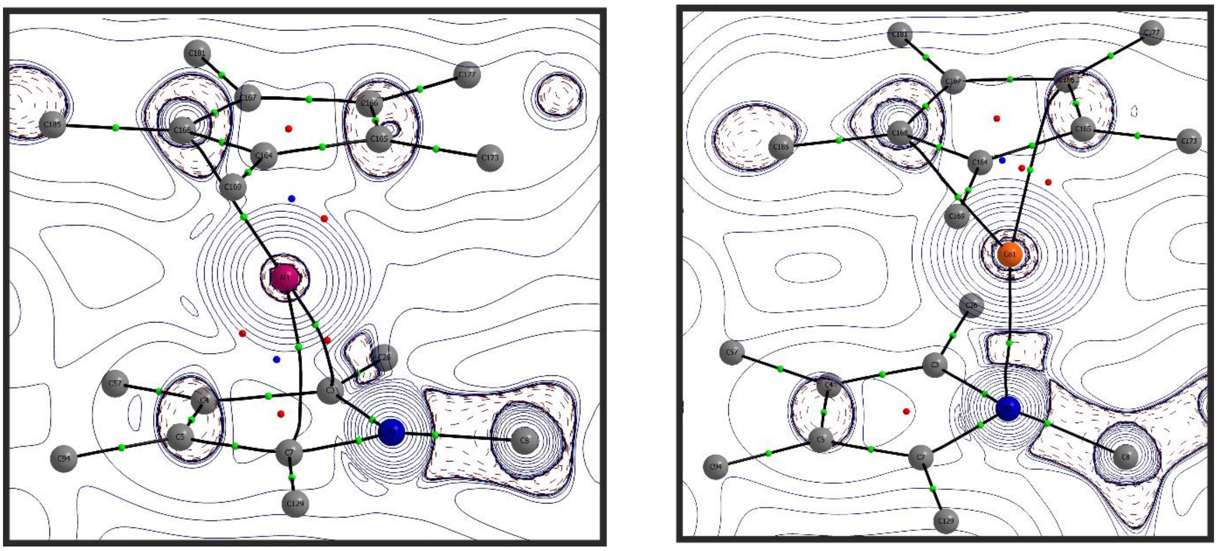

Figure SI AIMAll plots for molecular excerpts of the topological analysis of compound **1** (left) and **2** (right). In both cases a contour plot of the Laplacian of the electron density ($\nabla^2\rho(r)$) through the moleculars central plane (E-B-[C_β-C_β]$_{centroid}$) is also depicted. Bond critical points are depicted in green, ring critical points are depicted in red and cage critical points are depicted in blue. Blue lines indicate positive Laplacian (area of charge depletion), maroon dotted lines indicate negative Laplacian (area of charge concentration).

To shed further light onto the structure analysis of the aluminium sandwich complex further analyses were carried out. The results from topology analyses did not differ between wavefunctions obtained from BP86 or PBE0 functional calculations and no qualitative change between def-SVP basis sets and def2-TZVPP basis sets were observed. In all cases same CP and bonding path were found giving the same molecular graphs. We further investigated the parent all hydro substituted η^5,η^5-(C_4BH_5),(C_5H_5) Al complex. Structures have been optimised using both BP86 and PBE0 functional and def2-TZVPP basis sets. No imaginary frequencies were found confirming minimum structures. The geometries obtained are summarised in the following Figure.

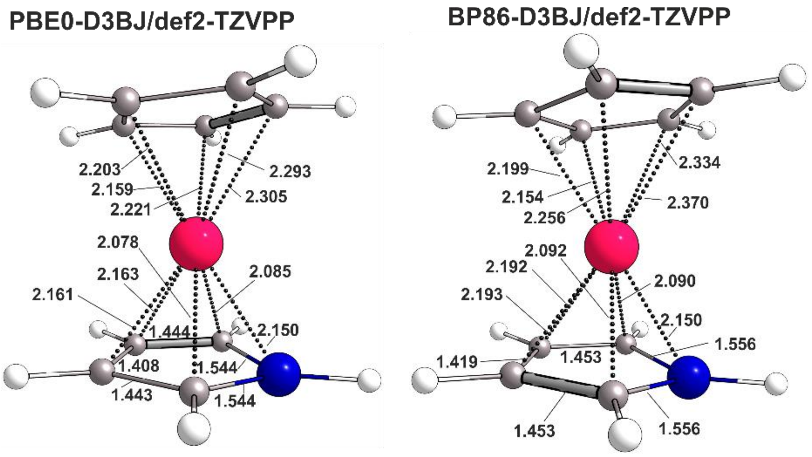

Figure SI Structural features of the optimised geometries for parent borole/Cp “aluminocenes”.

Some features of the QTAIM analyses for both calculations are depicted below. The isodensity surfaces show that electron density around the boron atom is significantly reduced when compared to the densities at C_α but also C_β.

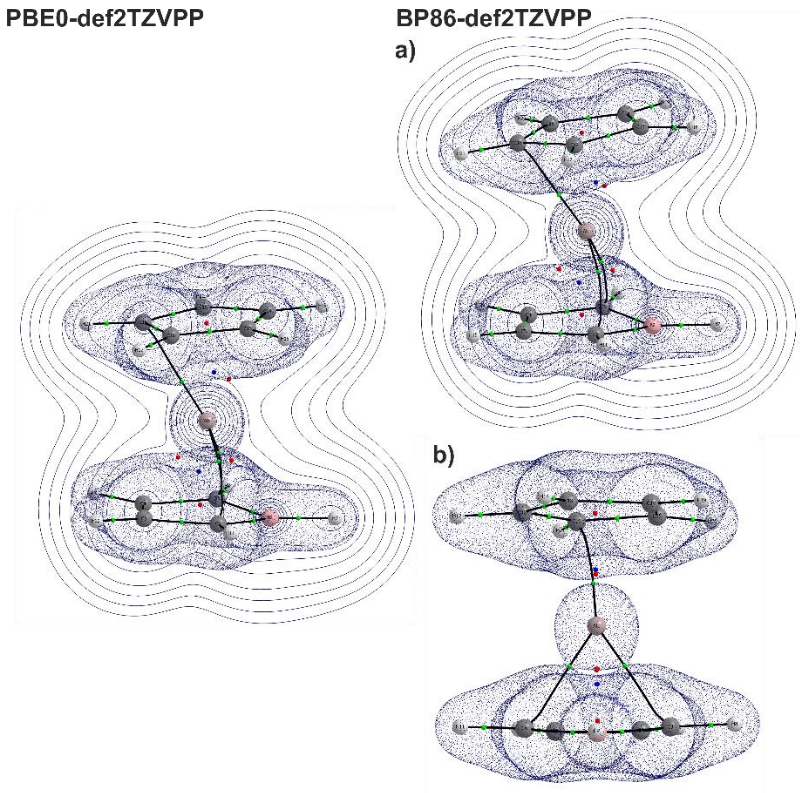

Electron density contour plot through the Al-B-(C_β-C_β)$_{ctr}$ plane.
Electron density isosurface at the Al-C_α BCP level (ρ = 0.063 a.u.)

Electron density contour plot through the Al-B-(C_β-C_β)$_{ctr}$ plane.
Electron density isosurface at the Al-C_α BCP level (ρ = 0.062 a.u.)
a) view from the side
b) view from the front

Figure SI AIMAll plots for $(C_4BH_5)Al(C_5H_5)$ from PBE0-def2TZVPP (left) and BP86-def2TZVPP calculations. BCP (green), RCP (red) and CCP (blue).

Bader charges for model complex $(C_4BH_5)Al(C_5H_5)$

		BP86	PBE0
	Al1	2,2460	2,3647
Borole	B2	1,6350	1,7559
	C3	-1,0462	-1,0989
	C4	-0,2460	-0,2645
	C5	-0,2455	-0,2631
	C6	-1,0455	-1,0998
	H7	-0,6122	-0,6447
	H8	0,0146	0,0122
	H9	0,0201	0,0190
	H10	0,0200	0,0193
	H11	0,0143	0,0116
	sum	-1,4914	-1,5531
Cp	C12	-0,2179	-0,2417
	C13	-0,1622	-0,1869
	C14	-0,1766	-0,1846
	C15	-0,2533	-0,2360
	C16	-0,2826	-0,2740
	H17	0,0596	0,0613
	H18	0,0583	0,0613
	H19	0,0583	0,0614
	H20	0,0610	0,0622
	H21	0,0634	0,0637
Cp	sum	-0,7919	-0,8133
Total	sum	-0,0373	-0,0017

NBO and NRT Analyses

NBO and NRT analyses were performed using NBO7 on a $[C_4BH_5]^{2-}$ structure optimised using ORCA BP86-D3BJ and def2-TZVPP basis set.[28]

XYZ-coordinates of optimised structures

Using ORCA4.1 RI-BP86-D3BJ-def2TZVP/J model chemistry

Optimised Structure of compound 1

Al	10.250973000	9.943841000	18.456661000
B	8.116703000	10.381779000	18.363243000
C	8.857187000	11.252515000	19.409685000
C	9.393961000	10.364258000	20.442762000
C	9.143172000	8.995364000	20.091128000
F	4.090155000	9.636041000	13.814464000
C	8.424707000	8.933112000	18.817162000
C	7.350734000	10.822064000	17.070577000
F	3.979189000	8.714718000	15.798701000
C	7.808550000	11.873266000	16.255945000
H	8.704220000	12.420729000	16.543891000
F	3.110931000	10.693111000	15.458905000
C	5.968429000	11.574593000	14.705025000
H	5.441071000	11.859338000	13.795622000
C	5.482006000	10.548371000	15.519078000
C	6.167426000	10.169206000	16.675841000
H	5.779673000	9.350365000	17.280668000
C	4.176212000	9.892752000	15.151760000
C	7.140674000	12.233220000	15.084551000
C	7.715465000	13.289908000	14.180616000
F	8.566878000	14.129640000	14.830850000
F	8.434733000	12.732088000	13.154739000
F	6.754776000	14.059776000	13.602121000
C	7.805515000	13.474604000	19.100477000
H	6.922820000	12.937433000	18.754887000
C	8.923298000	12.725570000	19.484955000
C	7.793404000	14.874820000	19.169012000
C	5.367343000	15.223863000	19.670032000
H	4.444885000	15.751214000	19.383259000
H	5.598258000	15.473466000	20.715831000
H	5.171736000	14.144260000	19.615532000
C	6.530732000	15.632682000	18.740547000
C	6.178033000	15.260514000	17.285245000
H	5.264924000	15.785842000	16.967592000
H	6.001636000	14.182385000	17.172741000
H	6.991840000	15.540290000	16.602226000
C	6.707724000	17.156040000	18.822990000
H	7.524641000	17.504916000	18.174488000
H	6.915529000	17.488097000	19.850695000
H	5.783811000	17.652603000	18.493514000
C	8.931536000	15.518632000	19.663815000
H	8.937173000	16.604451000	19.733045000
C	10.063850000	14.803250000	20.094365000
C	11.283537000	15.548432000	20.653924000
C	10.837069000	16.682234000	21.599484000
H	10.248824000	17.449687000	21.079415000
H	11.718257000	17.178854000	22.031858000
H	10.225435000	16.290014000	22.424102000
C	12.072816000	16.155173000	19.474198000
H	12.415188000	15.365958000	18.788701000
H	12.956087000	16.703101000	19.837288000
H	11.447734000	16.851926000	18.897810000
C	12.210487000	14.610133000	21.447338000
H	12.705482000	13.875582000	20.799615000
H	11.662520000	14.055417000	22.222187000
H	13.001093000	15.196718000	21.937151000
C	10.025943000	10.795974000	21.702124000
C	10.048835000	13.413459000	19.976767000
H	10.905531000	12.833675000	20.306925000
C	11.196737000	10.204403000	22.194624000
H	11.667678000	9.419710000	21.607234000
C	11.727303000	10.569818000	23.436910000
C	11.080884000	11.585232000	24.156060000
H	11.491917000	11.893566000	25.116901000
C	9.917131000	12.212710000	23.686660000
C	9.392342000	11.788376000	22.464050000
H	8.477954000	12.227836000	22.073783000
C	12.999187000	9.913741000	23.989340000
C	13.288102000	8.569075000	23.299400000
H	12.419124000	7.896317000	23.339353000
H	14.133631000	8.070849000	23.794945000
H	13.561296000	8.700657000	22.244357000
C	14.186225000	10.870160000	23.745438000
H	14.312474000	11.065566000	22.670456000
H	15.122045000	10.435795000	24.129654000
H	14.022692000	11.835727000	24.244712000
C	12.861978000	9.650421000	25.502462000
H	11.989526000	9.016751000	25.715202000
H	12.753182000	10.579034000	26.078167000
H	13.759240000	9.135329000	25.875842000
C	9.220771000	13.290858000	24.526747000
C	8.531939000	12.603335000	25.725563000
H	7.789445000	11.870514000	25.378587000
H	8.017687000	13.346323000	26.354303000
H	9.263621000	12.071457000	26.350318000
C	10.249098000	14.313212000	25.051370000
H	10.776542000	14.803676000	24.221655000
H	11.001591000	13.846932000	25.701430000
H	9.739000000	15.090367000	25.639510000
C	8.163365000	14.052988000	23.709528000
H	8.593365000	14.486861000	22.794771000
H	7.747379000	14.871061000	24.314997000
H	7.326746000	13.404698000	23.413948000
C	9.510043000	7.816264000	20.894989000
C	10.033972000	6.668751000	20.290957000
H	10.211138000	6.682053000	19.216950000
C	10.025972000	5.523860000	22.398540000
H	10.208645000	4.626645000	22.987778000
C	9.519163000	6.665653000	23.045651000
C	9.249562000	7.796107000	22.275123000
H	8.823852000	8.686055000	22.731312000
C	10.281401000	5.500508000	21.023534000
C	10.828884000	4.260268000	20.303978000
C	12.301544000	4.520434000	19.922699000
H	12.726725000	3.648861000	19.401985000
H	12.908118000	4.720230000	20.817863000
H	12.389038000	5.389600000	19.256688000
C	10.010558000	3.987072000	19.024500000
H	10.042027000	4.827310000	18.319260000
H	8.953955000	3.807363000	19.263329000
H	10.403336000	3.098805000	18.507621000
C	10.766699000	3.004689000	21.187670000
H	9.738561000	2.796085000	21.517644000
H	11.403337000	3.096745000	22.079281000
H	11.121583000	2.134066000	20.617645000
C	9.236568000	6.630220000	24.552602000
C	8.075127000	5.647720000	24.815352000
H	7.165392000	5.973566000	24.291101000
H	7.852943000	5.591665000	25.891950000
H	8.321003000	4.636099000	24.463302000
C	8.848789000	8.014879000	25.097056000
H	9.621539000	8.768565000	24.885322000
H	8.716685000	7.959504000	26.187203000
H	7.902151000	8.372105000	24.667380000
C	10.492024000	6.149035000	25.309284000
H	10.795160000	5.139478000	25.000592000
H	10.296189000	6.124354000	26.391716000
H	11.340805000	6.823968000	25.130532000
C	7.866031000	7.688223000	18.263112000
C	7.928211000	7.385624000	16.897286000
H	8.432280000	8.087698000	16.232512000
C	7.349520000	6.220698000	16.375478000
C	6.713379000	5.343323000	17.262886000
H	6.274221000	4.427811000	16.874934000
C	7.185374000	6.800064000	19.112687000
H	7.115219000	7.054815000	20.167869000
C	7.432412000	5.952732000	14.866667000
C	6.757799000	7.116484000	14.110464000
H	6.829358000	6.960704000	13.023441000
H	7.229643000	8.080351000	14.345848000
H	5.695600000	7.190343000	14.378501000
C	8.913408000	5.862699000	14.444331000
H	8.993032000	5.654967000	13.366535000
H	9.429645000	5.059826000	14.990686000
H	9.441823000	6.803983000	14.644981000
C	6.735028000	4.644445000	14.465878000
H	6.814084000	4.503125000	13.378355000
H	5.666400000	4.659824000	14.723738000
H	7.195605000	3.771560000	14.951257000
C	6.617337000	5.617591000	18.637552000
C	5.852758000	4.691859000	19.594966000
C	6.493541000	4.688710000	20.997780000
H	7.555371000	4.409155000	20.957807000
H	5.970757000	3.968475000	21.644090000
H	6.433262000	5.669991000	21.486041000
C	5.820680000	3.240966000	19.082357000
H	5.265442000	3.146415000	18.139101000
H	5.320443000	2.598353000	19.821317000
H	6.835711000	2.849067000	18.922051000
C	4.403454000	5.214799000	19.706567000
H	3.911282000	5.215799000	18.723424000
H	4.389841000	6.244305000	20.091637000
H	3.814654000	4.582215000	20.388935000
C	11.611807000	8.728132000	17.207623000
C	11.198916000	9.849204000	16.410119000
C	11.616426000	11.048643000	17.075297000
C	12.291917000	10.673716000	18.287290000
C	12.290435000	9.233951000	18.371142000
C	11.447089000	7.279964000	16.859542000
H	11.763679000	7.093585000	15.822813000
H	10.407338000	6.932557000	16.944407000
H	12.066257000	6.651953000	17.512071000
C	10.438207000	9.764924000	15.120794000
H	10.696759000	10.596789000	14.452902000
H	9.347849000	9.800131000	15.267140000
H	10.670973000	8.828936000	14.595149000
C	11.414647000	12.457329000	16.602534000
H	12.382062000	12.939331000	16.394072000
H	10.895310000	13.075316000	17.350267000
H	10.826977000	12.486676000	15.677840000

C	13.008865000	11.599136000	19.220602000
H	14.099420000	11.487401000	19.112787000
H	12.758025000	11.412438000	20.274931000
H	12.758890000	12.642300000	18.996216000
C	13.009626000	8.422386000	19.405646000
H	13.793693000	7.807918000	18.937282000
H	12.345066000	7.744487000	19.959245000
H	13.500303000	9.080351000	20.132679000

Optimised Structure of compound 2

Ga	10.214673000	10.254279000	17.451441000
B	8.281213000	10.297082000	18.356838000
C	8.804268000	11.213483000	19.568032000
C	9.172172000	10.346873000	20.586785000
C	8.933683000	8.937745000	20.228407000
F	3.501278000	8.441664000	16.830822000
C	8.405875000	8.816784000	18.953900000
C	7.165045000	10.650544000	17.286413000
F	3.014584000	10.167554000	18.080227000
C	7.385077000	11.387982000	16.108857000
H	8.378976000	11.791726000	15.908281000
F	2.544719000	10.198402000	15.941433000
C	5.080447000	11.110930000	15.406265000
H	4.285188000	11.274635000	14.681267000
C	4.839742000	10.389432000	16.573207000
C	5.868203000	10.153679000	17.493071000
H	5.660406000	9.555828000	18.381621000
C	3.477603000	9.807022000	16.849572000
C	6.368526000	11.608570000	15.179940000
C	6.639009000	12.344826000	13.894963000
F	7.899587000	12.856687000	13.837145000
F	6.498477000	11.527940000	12.807911000
F	5.777319000	13.383916000	13.707253000
C	8.104479000	13.488130000	18.845052000
H	7.265170000	13.016658000	18.335471000
C	8.993651000	12.669920000	19.555716000
C	8.258492000	14.882101000	18.793848000
C	5.834760000	15.490128000	18.571495000
H	5.093431000	16.072673000	18.003948000
H	5.788556000	15.803631000	19.624465000
H	5.543221000	14.432376000	18.521107000
C	7.248728000	15.715369000	17.994591000
C	7.275036000	15.255802000	16.521903000
H	6.555080000	15.832970000	15.922962000
H	7.011522000	14.194154000	16.432206000
H	8.275240000	15.393320000	16.086283000
C	7.560480000	17.218186000	18.043151000
H	8.549840000	17.442650000	17.618239000
H	7.529512000	17.607494000	19.071319000
H	6.812549000	17.768786000	17.454720000
C	9.331672000	15.452134000	19.483682000
H	9.465242000	16.532877000	19.462675000
C	10.247882000	14.667786000	20.211867000
C	11.394963000	15.343723000	20.973380000
C	10.801525000	16.350884000	21.981418000
H	10.222344000	17.136412000	21.477325000
H	11.603875000	16.837135000	22.556796000
H	10.130370000	15.842017000	22.687855000
C	12.303030000	16.092932000	19.976303000
H	12.745535000	15.393580000	19.251720000
H	13.123022000	16.600012000	20.507383000
H	11.742692000	16.851609000	19.412626000
C	12.250952000	14.331169000	21.751833000
H	12.755669000	13.623071000	21.079628000
H	11.652617000	13.749060000	22.467119000
H	13.029482000	14.864215000	22.316781000
C	9.805991000	10.711878000	21.870412000
C	10.072345000	13.288318000	20.223414000
H	10.773941000	12.658467000	20.760513000
C	11.018866000	10.123697000	22.236916000
H	11.467721000	9.400865000	21.557915000
C	11.640295000	10.418776000	23.456589000
C	11.013095000	11.336496000	24.303363000
H	11.470565000	11.571083000	25.262992000
C	9.798801000	11.962510000	23.963080000
C	9.203412000	11.630887000	22.744994000
H	8.262412000	12.084695000	22.440109000
C	12.977385000	9.748639000	23.793632000
C	14.057651000	10.322736000	22.851245000
H	13.809433000	10.123375000	21.799377000
H	15.037808000	9.867877000	23.061092000
H	14.144890000	11.411684000	22.975956000
C	13.406398000	10.005925000	25.245756000
H	13.574902000	11.075016000	25.439390000
H	14.349071000	9.479310000	25.452982000
H	12.652561000	9.641263000	25.958745000
C	12.871863000	8.223639000	23.579690000
H	12.602276000	7.966475000	22.546967000
H	12.103818000	7.784852000	24.230926000
H	13.834833000	7.742423000	23.807770000
C	9.152179000	12.949698000	24.943056000
C	8.638699000	12.162430000	26.167843000
H	7.881400000	11.424519000	25.866869000
H	8.183576000	12.844317000	26.902412000
H	9.457017000	11.620699000	26.662692000
C	10.189790000	13.992200000	25.408644000
H	10.594380000	14.553446000	24.555032000
H	11.033552000	13.526992000	25.935644000
H	9.720168000	14.708837000	26.098786000
C	7.974057000	13.701590000	24.304028000
H	8.283861000	14.235997000	23.393864000
H	7.576491000	14.440018000	25.014975000
H	7.151286000	13.023450000	24.037183000
C	9.278372000	7.836102000	21.146030000
C	10.045820000	6.750650000	20.703240000
H	10.331861000	6.720281000	19.650979000
C	9.998278000	5.789238000	22.904514000
H	10.288487000	4.998920000	23.591827000
C	9.217622000	6.852247000	23.380515000
C	8.867388000	7.866197000	22.484435000
H	8.267158000	8.710588000	22.822316000
C	10.418181000	5.716878000	21.567196000
C	11.250098000	4.547952000	21.024147000
C	12.550016000	5.088835000	20.393031000
H	13.168962000	4.258255000	20.021463000
H	13.137125000	5.654521000	21.130949000
H	12.341468000	5.754442000	19.544688000
C	10.424939000	3.813650000	19.946399000
H	10.110238000	4.494371000	19.143307000
H	9.516516000	3.380617000	20.388308000
H	11.011632000	2.997285000	19.498122000
C	11.630431000	3.541410000	22.119955000
H	10.741839000	3.090265000	22.584193000
H	12.232385000	4.011381000	22.911306000
H	12.226524000	2.727453000	21.682781000
C	8.735809000	6.945536000	24.833415000
C	7.192490000	6.901940000	24.845918000
H	6.764784000	7.735581000	24.272413000
H	6.814720000	6.968555000	25.877531000
H	6.826237000	5.964749000	24.402389000
C	9.213833000	8.278668000	25.447078000
H	10.311286000	8.323785000	25.481135000
H	8.833841000	8.383990000	26.474457000
H	8.872848000	9.145526000	24.866715000
C	9.265092000	5.794074000	25.700504000
H	8.917993000	4.815753000	25.337512000
H	8.904644000	5.913396000	26.732381000
H	10.364540000	5.780930000	25.729599000
C	7.879201000	7.607894000	18.320693000
C	7.814950000	7.512514000	16.922173000
H	8.232922000	8.327238000	16.328358000
C	7.198435000	6.432939000	16.277405000
C	6.667166000	5.410735000	17.069159000
H	6.180553000	4.563306000	16.588970000
C	7.312269000	6.561776000	19.079960000
H	7.325625000	6.647588000	20.162560000
C	7.091524000	6.441317000	14.747119000
C	6.294500000	7.691066000	14.313551000
H	6.202886000	7.729025000	13.217588000
H	6.784944000	8.616499000	14.644030000
H	5.285128000	7.676569000	14.746741000
C	8.504860000	6.504340000	14.134361000
H	8.450547000	6.520414000	13.035387000
H	9.105659000	5.635565000	14.440569000
H	9.030411000	7.413513000	14.457691000
C	6.380320000	5.194426000	14.202160000
H	6.329622000	5.247796000	13.105053000
H	5.350709000	5.119080000	14.580033000
H	6.915002000	4.270971000	14.468844000
C	6.719246000	5.457155000	18.475778000
C	6.083661000	4.331218000	19.301278000
C	6.363872000	4.489913000	20.805143000
H	7.441728000	4.529836000	21.019501000
H	5.938850000	3.635269000	21.351351000
H	5.906552000	5.403144000	21.211512000
C	6.644764000	2.968783000	18.843775000
H	6.422075000	2.770247000	17.786615000
H	6.200054000	2.156258000	19.438000000
H	7.735562000	2.931760000	18.970601000
C	4.556069000	4.354036000	19.078550000
H	4.305029000	4.211701000	18.018123000
H	4.131783000	5.316710000	19.397430000
H	4.070331000	3.552410000	19.655926000
C	11.855654000	8.938203000	16.608437000
C	11.611548000	9.983028000	15.650470000
C	11.913105000	11.243381000	16.279290000
C	12.357692000	10.977125000	17.620064000
C	12.317003000	9.553168000	17.826380000
C	11.698487000	7.463019000	16.402218000
H	11.066078000	7.238284000	15.535464000
H	11.237277000	6.979956000	17.275112000
H	12.676129000	6.983002000	16.236891000
C	11.150930000	9.803920000	14.236916000
H	10.482558000	10.615001000	13.917699000
H	10.613125000	8.857124000	14.101888000
H	12.007963000	9.795564000	13.544802000
C	11.809181000	12.597610000	15.647423000
H	12.771124000	12.901448000	15.204395000
H	11.528679000	13.364076000	16.382568000
H	11.060623000	12.614396000	14.844396000
C	12.828952000	11.993636000	18.611023000

H	13.906470000	12.188566000	18.488921000
H	12.666487000	11.654400000	19.642341000
H	12.298457000	12.947411000	18.497036000
C	12.764559000	8.825260000	19.054378000
H	13.743254000	8.347034000	18.892173000
H	12.059539000	8.038787000	19.353209000
H	12.869553000	9.511761000	19.902651000

Optimised Structure of (C_4BH_5)Al(C_5H_5) (PBE0-def2TZVPP)

Al	10.292815000	10.014749000	18.617609000
B	8.246237000	10.669300000	18.698756000
C	9.100034000	11.161029000	19.886871000
C	9.536778000	10.012617000	20.644800000
C	9.134608000	8.825974000	20.002398000
C	8.416193000	9.137841000	18.789855000
H	7.732278000	11.313008000	17.833863000
H	9.343101000	12.165326000	20.210651000
H	10.154652000	10.043267000	21.533093000
H	9.406129000	7.833405000	20.338824000
H	8.053319000	8.354044000	18.137744000
C	11.827541000	8.728893000	17.698298000
C	11.351094000	9.537507000	16.639574000
C	11.631101000	10.883243000	16.954392000
C	12.280279000	10.919194000	18.209853000
C	12.407443000	9.583023000	18.674345000
H	11.744051000	7.655743000	17.770841000
H	10.806393000	9.188138000	15.776297000
H	11.335223000	11.743284000	16.374383000
H	12.599483000	11.802964000	18.740009000
H	12.854136000	9.270668000	19.605342000

Optimised Structure of (C_4BH_5)Al(C_5H_5) (BP86-def2TZVPP)

Al	10.272210000	9.978443000	18.600052000
B	8.161743000	10.386534000	18.599129000
C	8.958963000	11.234220000	19.632526000
C	9.524555000	10.340114000	20.629064000
C	9.262141000	8.990264000	20.277665000
C	8.511892000	8.934765000	19.034458000
H	7.566661000	10.755523000	17.626211000
H	9.081225000	12.313721000	19.710137000
H	10.140118000	10.643778000	21.474796000
H	9.650936000	8.129765000	20.820537000
H	8.239727000	7.981784000	18.582760000
C	11.698614000	8.743647000	17.363027000
C	11.388502000	9.842721000	16.514291000
C	11.783220000	11.037277000	17.170362000
C	12.346708000	10.689141000	18.433286000
C	12.301611000	9.259141000	18.554792000
H	11.487050000	7.698168000	17.163138000
H	10.861452000	9.781680000	15.567630000
H	11.621255000	12.047582000	16.808616000
H	12.732402000	11.377658000	19.178298000
H	12.661904000	8.677293000	19.397024000

Optimised Structure of $(C_4BH_5)^{2-}$ (BP86-def2TZVPP)

B	8.248341000	10.379542000	18.543611000
C	8.928423000	11.251470000	19.621769000
C	9.475150000	10.366644000	20.615006000
C	9.210008000	9.010226000	20.274590000
C	8.475586000	8.934950000	19.040397000
H	7.655124000	10.753444000	17.514013000
H	9.036226000	12.348022000	19.720525000
H	10.028801000	10.671792000	21.522982000
H	9.536371000	8.150879000	20.890279000
H	8.178562000	7.956720000	18.617730000

Literature

[1] R. K. Harris, E. D. Becker, S. M. Cabral de Menezes, R. Goodfellow, P. Granger, *Pure Appl. Chem.* **2001**, *73*, 1795-1818.

[2] T. Heitkemper, C. P. Sindlinger, *Chem. Eur. J.* **2019**, *25*, 6628-6637.

[3] Z. J. Tonzetich, R. Eisenberg, *Inorg. Chim. Acta* **2003**, *345*, 340-344.

[4] a) M. Schormann, K. S. Klimek, H. Hatop, S. P. Varkey, H. W. Roesky, C. Lehmann, C. Röpken, R. Herbst-Irmer, M. Noltemeyer, *J. Solid State Chem.* **2001**, *162*, 225-236; b) S. Schulz, H. W. Roesky, H. J. Koch, G. M. Sheldrick, D. Stalke, A. Kuhn, *Angew. Chem. Int. Ed. Engl.* **1993**, *32*, 1729-1731.

[5] P. Jutzi, B. Neumann, G. Reumann, H.-G. Stammler, *Organometallics* **1998**, *17*, 1305-1314.

[6] SAINTv8.30C, Bruker AXS, WI, USA, Madison, **2013**.

[7] L. Krause, R. Herbst-Irmer, G. M. Sheldrick, D. Stalke, *J. Appl. Crystallogr.* **2015**, *48*, 3-10.

[8] SADABS, G. M. Sheldrick, University of Göttingen, Göttingen, **2008**.

[9] G. M. Sheldrick, *Acta Crystallogr.* **2015**, *A71*, 3.

[10] G. M. Sheldrick, *Acta Crystallogr.* **2015**, *C71*, 3.

[11] C. B. Hübschle, G. M. Sheldrick, B. Dittrich, *J. Appl. Crystallogr.* **2011**, *44*, 1281-1284.

[12] D. Kratzert, I. Krossing, *J. Appl. Crystallogr.* **2018**, *51*, 928-934.

[13] A. Spek, *Acta Crystallographica Section C* **2015**, *71*, 9-18.

[14] M. J. Frisch, G. W. Trucks, H. B. Schlegel, G. E. Scuseria, M. A. Robb, J. R. Cheeseman, G. Scalmani, V. Barone, B. Mennucci, G. A. Petersson, H. Nakatsuji, M. Caricato, X. Li, H. P. Hratchian, A. F. Izmaylov, J. Bloino, G. Zheng, J. L. Sonnenberg, M. Hada, M. Ehara, K. Toyota, R. Fukuda, J. Hasegawa, M. Ishida, T. Nakajima, Y. Honda, O. Kitao, H. Nakai, T. Vreven, J. A. Montgomery Jr., J. E. Peralta, F. Ogliaro, M. J. Bearpark, J. Heyd, E. N. Brothers, K. N. Kudin, V. N. Staroverov, R. Kobayashi, J. Normand, K. Raghavachari, A. P. Rendell, J. C. Burant, S. S. Iyengar, J. Tomasi, M. Cossi, N. Rega, N. J. Millam, M. Klene, J. E. Knox, J. B. Cross, V. Bakken, C. Adamo, J. Jaramillo, R. Gomperts, R. E. Stratmann, O. Yazyev, A. J. Austin, R. Cammi, C. Pomelli, J. W. Ochterski, R. L. Martin, K. Morokuma, V. G. Zakrzewski, G. A. Voth, P. Salvador, J. J. Dannenberg, S. Dapprich, A. D. Daniels, Ö. Farkas, J. B. Foresman, J. V. Ortiz, J. Cioslowski, D. J. Fox, Gaussian, Inc., Wallingford, CT, USA, **2009**.

[15] a) A. D. Becke, *Phys. Rev. A* **1988**, *38*, 3098-3100; b) J. P. Perdew, W. Yue, *Phys. Rev. B* **1986**, *33*, 8800-8802.

[16] S. Grimme, S. Ehrlich, L. Goerigk, *J. Comput. Chem.* **2011**, *32*, 1456-1465.

[17] a) A. Schäfer, C. Huber, R. Ahlrichs, *J. Chem. Phys.* **1994**, *100*, 5829-5835; b) F. Weigend, R. Ahlrichs, *Phys. Chem. Chem. Phys.* **2005**, *7*, 3297-3305.

[18] a) F. Neese, *Wiley Interdiscip. Rev. Comput. Mol. Sci.* **2012**, *2*, 73-78; b) F. Neese, *Wiley Interdiscip. Rev. Comput. Mol. Sci.* **2018**, *8*, e1327.

[19] J. P. Perdew, K. Burke, M. Ernzerhof, *Phys. Rev. Lett.* **1996**, *77*, 3865-3868.

[20] K. Eichkorn, F. Weigend, O. Treutler, R. Ahlrichs, *Theor. Chem. Acc.* **1997**, *97*, 119-124.

[21] R. W. Schurko, I. Hung, C. L. B. Macdonald, A. H. Cowley, *J. Am. Chem. Soc.* **2002**, *124*, 13204-13214.

[22] a) R. Benn, A. Rufińska, H. Lehmkuhl, E. Janssen, C. Krüger, *Angew. Chem.* **1983**, *95*, 808-809; b) R. Benn, E. Janssen, H. Lehmkuhl, A. Rufínska, *J. Organomet. Chem.* **1987**, *333*, 155-168.

[23] R. W. Schurko, I. Hung, S. Schauff, C. L. B. Macdonald, A. H. Cowley, *J. Phys. Chem. A* **2002**, *106*, 10096-10107.

[24] ChemCraft, G. A. Zhurko, Version 1.7., **2014**.

[25] R. F. W. Bader, *Atoms in Molecules A Quantum Theory*, Oxford University Press, Oxford, **1990**.

[26] T. Lu, F. Chen, *J. Comput. Chem.* **2012**, *33*, 580-592.

[27] AIMall, T. A. Keith, TK Gristmill Software, Overland Parks KS USA, **2019**.

[28] a) F. Weinhold, C. R. Landis, E. D. Glendening, *J. Am. Chem. Soc.* **2019**, *141*, 4156-4166. ; b) NBO7, E. D. Glendening, J. K. Badenhoop, A. E. Reed, J. E. Carpenter, J. A. Bohmann, C. M. Morales, P. Karafiloglou, C. R. Landis, F. Weinhold, Theoretical Chemical Institute, University of Wisconsin Madison, **2018**.
